# Lipid‐based nanoparticles as drug delivery systems for cancer immunotherapy

**DOI:** 10.1002/mco2.339

**Published:** 2023-08-07

**Authors:** Yang Hao, Zhonghao Ji, Hengzong Zhou, Dongrun Wu, Zili Gu, Dongxu Wang, Peter ten Dijke

**Affiliations:** ^1^ Department of Laboratory Animals College of Animal Sciences Jilin University Changchun China; ^2^ Department of Basic Medicine Changzhi Medical College Changzhi China; ^3^ Department of Cell and Chemical Biology and Oncode Institute Leiden University Medical Center Leiden The Netherlands; ^4^ Departure of Philosophy, Faculty of Humanities Leiden University Leiden The Netherlands; ^5^ Department of Radiology Leiden University Medical Center Leiden The Netherlands

**Keywords:** lipid‐based nanoparticles, cancer immunotherapy, stimulator of interferon genes, type I interferon, tumor microenvironment

## Abstract

Immune checkpoint inhibitors (ICIs) have shown remarkable success in cancer treatment. However, in cancer patients without sufficient antitumor immunity, numerous data indicate that blocking the negative signals elicited by immune checkpoints is ineffective. Drugs that stimulate immune activation‐related pathways are emerging as another route for improving immunotherapy. In addition, the development of nanotechnology presents a promising platform for tissue and cell type‐specific delivery and improved uptake of immunomodulatory agents, ultimately leading to enhanced cancer immunotherapy and reduced side effects. In this review, we summarize and discuss the latest developments in nanoparticles (NPs) for cancer immuno‐oncology therapy with a focus on lipid‐based NPs (lipid‐NPs), including the characteristics and advantages of various types. Using the agonists targeting stimulation of the interferon genes (STING) transmembrane protein as an exemplar, we review the potential of various lipid‐NPs to augment STING agonist therapy. Furthermore, we present recent findings and underlying mechanisms on how STING pathway activation fosters antitumor immunity and regulates the tumor microenvironment and provide a summary of the distinct STING agonists in preclinical studies and clinical trials. Ultimately, we conduct a critical assessment of the obstacles and future directions in the utilization of lipid‐NPs to enhance cancer immunotherapy.

## Graphical abstract

Cancer is a prevalent and lethal ailment that has a considerable social and economic impact globally. Despite notable progress in recent years, immunotherapy poses a challenge due to the limited response of a subset of cancer patients in the clinic. Lipid‐based nanoparticles (lipid‐NPs) have garnered significant interest for their capacity to enhance the effectiveness of cancer immunotherapy while minimizing toxic side effects. This review provides a summary of the physicochemical properties, applications, advantages, and disadvantages of various lipid‐NP types. Moreover, we provide an overview on how lipid‐NPs may broaden the therapeutic scope of cancer immunotherapy, using as example how lipid‐based NPs delivery of agonists targeting stimulators of interferon genes augments immunotherapy.

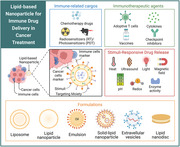

## 1 INTRODUCTION

Cancer represents a significant public health concern and remains the primary cause of mortality worldwide. Contemporary therapeutic modalities, encompassing conventional interventions such as surgery, chemotherapy, and radiotherapy, as well as novel targeted and photodynamic therapy (PDT), have yielded notable progress in the management of cancer. Notably, the emergence of immunotherapy that attempts to enhance or restore the host's immune system to destroy tumor cells has engendered renewed optimism for cancer patients.^1^ Despite the great progress made in recent years, the clinical success of immunotherapeutic drugs has been impeded by various challenges, including primary resistance in that majority of cancers patients do not respond to immunotherapy, immune‐related severe side effects, adaptive resistance resulting from immune escape mechanisms, and acquired resistance leading to cancer recurrence and metastasis.^2^ In light of these limitations, the integration of nanoparticles (NPs) in immunotherapy has remarkable potential, especially in the following aspects: antigen delivery and tumor antigen specificity, delivery of immunomodulators, modulation of the tumor microenvironment (TME), and combination therapy strategies.^3^

**FIGURE 1:**
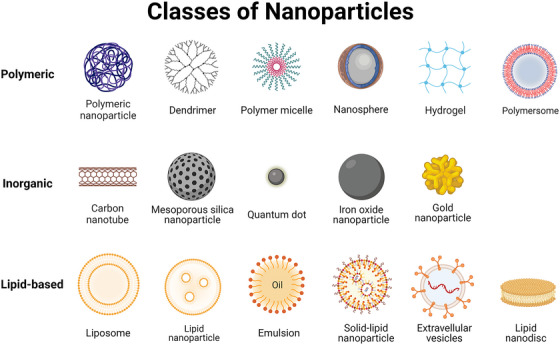
An overview of commonly used nanoparticles includes polymeric nanoparticles, inorganic nanoparticles and lipid‐based nanoparticles. Figure created with biorender.com.

NPs, typically ranging from 100 to 1000 nm in diameter, are composed of various materials with distinct surface modifications and physicochemical properties. Figure 1 presents a schematic representation of the commonly utilized NP types, which can be categorized into inorganic NPs, comprising metals such as gold and silver or silicon colloids, and organic NPs, consisting of lipids, sugars, and biodegradable polymers.^4^ While poly (lactic‐co‐glycolic acid) (PLGA) NPs, hydrogels, micelles, and metal NPs have been investigated as potential carriers for anti‐cancer drugs in preclinical studies, only a restricted number of nanomedicines utilizing these formulations have been granted approval by the United States Food and Drug Administration (US FDA) for cancer treatment.^5^ An instance of this is Abraxane®, a polymeric NP with an albumin base (with an approximate size of 130 nm) that is commercially available for the administration of chemotherapeutic paclitaxel. It was sanctioned in 2005 for the management of various oncological indications, including breast cancer, non‐small cell lung cancer (NSCLC), pancreatic cancer, and gastric cancer.^6^ Furthermore, polymeric micelle‐based NP formulations have been developed as a delivery system for paclitaxel or Docetaxel (Genexol®, Nanoxel®, and Paclical®) to treat advanced cancer types.^7^, ^8^, ^9^, ^10^ Herein, we mainly focused on liposomal formulations as delivery platforms in cancer immunotherapy. Lipid‐based nanomaterials (referred to lipid‐NPs), are the most commonly utilized class in clinical settings approved by the US FDA, owing to their unique properties such as formulation simplicity, excellent biocompatibility and safety, and drug loading capacity.^11^ Notably, liposomal drugs, such as Doxil®, Myocet®, DaunoXome®, Onivyde®, Vyxeos®, Marqibo®, DepoCyt®, among others, have achieved significant success since their introduction to the market because of effective payload loading efficiency, low toxicity, less immunogenicity, as well as mass producibility and better cost effectiveness.^12^ Additionally, lipid‐NPs have demonstrated to serve as delivery systems for immune‐related drugs, which can be loaded with tumor‐associated antigens (TAAs), low‐dose chemotherapeutic agents, photosensitizers/photothermal agents, or other immune adjuvants or antibodies. Lipid‐NPs show the potential to enhance immunotherapeutic drug efficacy, mitigate toxicity, and overcome tumor‐driven immune resistance.^13^

The primary objective of immunotherapy is to initiate immune response cascades through the innate immune system, which involves the identification of pattern recognition receptors (PRRs), including pathogen‐associated molecular patterns (PAMPs) such as microbial nucleic acids and lipopolysaccharide (LPS), as well as damage‐associated molecular patterns (DAMPs) such as host DNA.^14^, ^15^ The stimulator of interferon genes (STING) protein, also referred to as TMEM173, MITA, or MPYS, is a recently discovered PRR that recognizes cyclic dinucleotides (CDNs) and activates antigen‐presenting cells (APCs). While initially identified as activated in response to viral infections, subsequent research has demonstrated that the STING pathway also responds to a variety of intra‐ and extracellular stimuli, including DNA damage, metabolic abnormalities, and abnormal cell death. Targeting the STING signaling pathway has emerged as a viable therapeutic strategy in tumor therapy.^16^ Recent studies have demonstrated that the activation of the STING pathway by agonists can elicit downstream immune responses, such as the production of interferons (IFNs) and proinflammatory cytokines, and the activation of natural killer (NK) cells and T cells, ultimately leading to the mitigation of cancer progression.^17^

Although preclinical studies have demonstrated the effective antitumor effects of multiple STING agonists, their potential therapeutic efficacy in clinical settings is limited by factors such as low cytoplasmic delivery rate, rapid immune clearance rate, noncellular targetability, and systemic inflammatory toxicity. To address these limitations, NPs have been proposed as delivery vehicles for STING agonists, as they can increase stability, prolong blood circulation time, enhance tumor targeting, and promote intracellular uptake thereby maximizing the antitumor immune effect and reducing adverse effects.^18^ This approach maximizes the antitumor immune effect while simultaneously minimizing adverse effects.^19^ As such, in this review, STING agonists were chosen as the prototypical immunotherapeutic agents to examine the application of NPs for their delivery, with the aim of enhancing the efficacy and safety of intracellular delivery.

This review aims to provide a comprehensive introduction of the diverse lipid‐NPs utilized in cancer immunotherapy, including their characteristics, and applications, advantages, and disadvantages. Furthermore, the review will explore the activation of STING pathway in promoting antitumor immunity and regulating the TME. Additionally, we will summarize the latest therapeutic interventions targeting the STING pathway in clinical trials. Finally, the potential and challenges of utilizing lipid‐based NPs to deliver STING agonists for cancer treatment will be critically evaluated and discussed.

## 2 LIPID‐BASED NPs AS DRUG DELIVERY SYSTEM IN CANCER IMMUNOTHERAPY

Lipid‐NPs, encompassing liposomes, lipid nanoparticles (LNPs), nanoemulsions (NEs), solid lipid nanoparticles (SLNs) and nanostructured lipid carriers (NLCs), extracellular vesicles (EVs), and lipid polymer hybrid nanoparticles (LPHNPs), and so on, exhibit significant potential for delivering therapies and other applications in theragnostic, cosmetics, and nutrition.^20^ The advantages and disadvantages of various lipid‐NPs as drug delivery systems are listed in Table 1.

**TABLE 1:** Summary of advantages and disadvantages of main lipid‐NPs.

Particle type	Advantages	Disadvantages
Liposomes	Payload flexibility for hydrophilic and hydrophobic cargo Good biocompatible and biodegradable Drug protection Reduced systemic toxicity Possible surface modification	Poor stability Poor drug‐loading efficiency for hydrophobic cargo Problematic immunocompatibility Toxicity of organic solvent Limited storage conditions Limited targeting Possible fast clearance Drug leakage Relative high production costs
Cationic lipid nanoparticles (LNPs)	Payload flexibility for hydrophilic and hydrophobic cargo and mRNA products High drug loading efficiency Good biocompatible and biodegradable Drug protection Reduced systemic toxicity Possible surface modification Controlled release	Poor stability Toxicity of cationic lipids
Extracellular vesicles (EVs)	Payload flexibility for hydrophilic and hydrophobic cargo and mRNA products Good biocompatible and biodegradable Inherited biological and immunological characteristics No toxicity Immunological escape Possible surface modification Ability to cross various biological barrier	Poor stability Poor drug‐loading efficiency Heterogeneous physicochemical properties Possible fast clearance No standardized manufacturing methods Difficult to scale‐up and control batch quality
Solid lipid nanoparticles (SLNs)	Payload flexibility for hydrophilic and hydrophobic cargo Good drug entrapment efficiency for hydrophobic cargo Good biocompatible and biodegradable Drug protection Low systemic toxicity Possible surface modification High stability Easy sterilization and scale‐up process Relative low production costs	Poor drug‐loading efficiency Gelling tendency Possible drug leakage during storage Uneven drug release Difficult to cross biological barriers
Nanostructured lipid carriers (NLCs)	All SLN's advantages High drug‐loading efficiency High stability and fewer drug lost during storage Sustainable drug release	Complex optimization process of solid/liquid lipids Gelling tendency Difficult to cross biological barriers
Nanoemulsion (NE)	Payload flexibility for hydrophilic and hydrophobic cargo Good drug entrapment efficiency for hydrophilic cargo Good stability Good biocompatible and biodegradable Greater absorption by organisms Easy preparation and easy drug loading Relative low production costs	Poor drug‐loading efficiency Toxicity of emulsifier Relative fewer surface modification possibility
Lipid polymer hybrid nanoparticles (LPHNPs)	Payload flexibility for hydrophilic and hydrophobic cargo and mRNA products Low systemic toxicity Drug protection High encapsulation efficiency (polymer core) Precise control of nanoparticles (NPs) physicochemical properties (polymer shell) Easy surface modification (polymer shell) Good biocompatible and biodegradable (lipid shell) High stability (polymer shell)	Poor drug‐loading efficiency (polymer core) Toxicity of cationic lipids (core–shell‐type hollow LPHNPs) Possibility for aggregation and toxicity (polymer shell) Difficult to scale‐up and high production costs (Polymer shell) Difficult to control batch quality (engineered LPHNPs)

**FIGURE 2:**
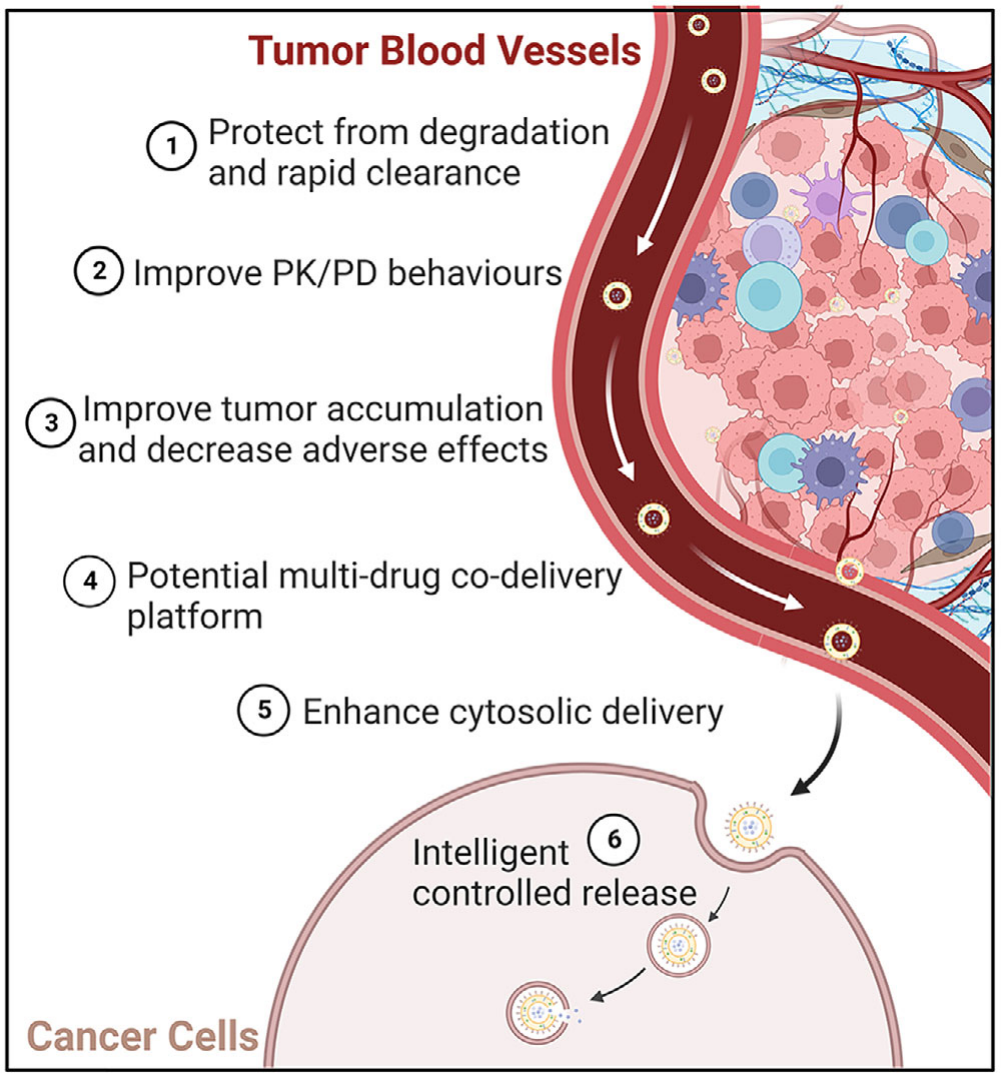
The utilization of lipid‐based nanomaterials in the delivery of immunotherapeutic agents, overcoming challenges, and improving therapeutic efficiency. PK/PD behaviours: pharmacokinetic‐pharmacodynamic behaviours. Figure was created with help of biorender.com.

Lipid‐NPs possess favorable safety, biocompatibility, degradability, and low toxicity due to their structural characteristics, which resemble those of the plasma membrane of human cells.^20^ Additionally, lipid‐NPs can serve as a delivery platform to protect encapsulated cargo from degradation and aggregation during circulation, thereby improving the pharmacokinetic attributes and physicochemical stability of immune agents or combined intervention drugs.^5^ Furthermore, lipid‐NPs are widely held to improve the delivery of cargos to tumors by two ways, namely passive and active targeting thereby enhancing intratumor aggregation and reducing ineffective systemic distribution of encapsulated cargos, ultimately resulting in a reduction of unexpected side effects.^21^ Passive targeting is also known as the enhanced permeability and retention (EPR) effect, which allows lipid‐NPs to leak preferentially into tumors through permeable tumor vessels and to enable efficient retention within the TME due to reduced lymphatic drainage.^22^ Active targeting facilitates the uptake of lipid‐NPs by specific cell types and other compositions in the TME, promoting the cellular uptake and efficiency of therapeutic agents.^23^ This can be achieved through the decoration of the lipid‐NPs surfaces with various targeting moieties such as tumor‐specific ligands (e.g., folate, transferrin, granulocyte–macrophage colony‐stimulating factor [GM‐CSF], etc.), peptides (e.g., RGD [Arg‐Gly‐Asp tripeptide] targeting cellular adhesion molecules, NGR [Asn‐Gly‐Arg tripeptide] targeting aminopeptidase N [CD13], etc.), or monoclonal antibodies (mAbs) (e.g., antivascular endothelial growth factor [VEGF]R, anti‐ERB‐B2 receptor tyrosine kinase 2, anticarcinoembryonic antigen, antimucin 1, anti‐CD33, etc.) to enhance their targeting properties. In this way, intratumor aggregation is enhanced and ineffective systemic distribution of encapsulated cargo is reduced, ultimately resulting in the reduction of unexpected side effects.^21^ Lipid‐NPs can also selectively target specific immune cells and other compositions in the TME, promoting the cellular uptake of therapeutic agents in APCs or T cells, thereby increasing their immunity‐boosting efficiency. Additionally, lipid‐NPs can achieve controlled drug release in both temporal and spatial dimensions through appropriate design such as magnetic field control, photosensitive control, PH or temperature responsiveness, and chemical substance control.^24^ (Detailed descriptions will be given in the Section 2.2.) To date, lipid‐NPs have been extensively studied as carriers for a diverse range of immunotherapeutic agents (e.g., antigens, antibodies, cells, recombinant proteins, and small molecular chemotherapeutic drugs).^24^, ^25^, ^26^, ^27^, ^28^ In particular, lipid‐based NPs play a crucial role in enhancing the safety and efficacy of immunotherapy (Figure 2).

### 2.1 General introductions of the early version lipid‐NPs: Liposomes

**FIGURE 3:**
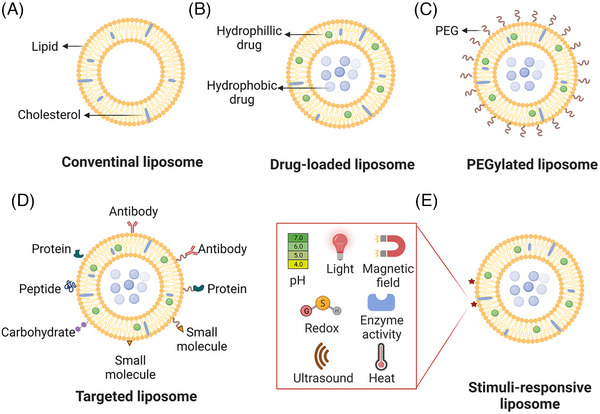
Lipid‐based nanomaterials for immunotherapeutic agents. (A) General schematic illustration of conventional liposomes as spherical vesicles based on a hydrophilic core of phospholipid bilayers and reinforced by cholesterol. (B) Schematic that demonstrates liposomal systems for the encapsulation of hydrophobic drugs into the bilayer membrane and the entrapment of hydrophilic drugs in the aqueous center. (C) Schematic representation of polyethylene glycol (PEG)‐coated liposomes for long circulation. (D) Schematic of multimolecule‐modified liposomes for specific targeting. (E) Schematic of multistimulus‐responsive liposomes for intelligent release. Figure was created with biorender.com.

Liposomes are the archetype version of lipid‐NPs and are defined as sphere‐like vesicles that consist of a hydrophilic core of phospholipid (PL) bilayers with different sizes.^29^, ^30^ They have the capacity to enclose aqueous drugs within the aqueous core of the vesicle and trap lipophilic drugs in the hydrocarbon chain region of the bilayer, even for macromolecular drugs.^31^ Typical liposomes are composed of PLs and contain sterol most often cholesterol to render an optimized structure and improved stability (Figure 3).^32^ In addition, it has been observed that the use of cholesterol derivatives, bacterial sterols, phytosterols, and so on can also play an important role in maintaining the mobility, permeability, and stability of PLs.^33^ The structure and physicochemical properties (e.g., size, numbers of concentric bilayers, and encapsulation ability) of liposomes depend strongly on their preparation route. The film hydration method and reverse phase evaporation represent the traditional liposome preparation technique (Table 2).^34^, ^35^, ^36^ According to the particle size and lamellarity, liposomes can be classified into unilamellar vesicles (ULVs; one bilayer), oligolamellar vesicles (OLVs; 2–5 bilayers, 100–1000 nm), and multilamellar vesicles (MVLs; >bilayers, >500 nm). The ULV can be further divided into three subclasses based on the particle size, including small unilamellar vesicles (SUVs; diameters 10–100 nm), large unilamellar vesicles (LUVs; diameters >100 nm), and giant unilamellar vesicles (GUVs; diameters >1000 nm).^37^ Both SUVs and MLVs can be prepared by controllably adjusting the operating parameters during the film hydration method, especially the hydration conditions, stirring speed, extrusion, and sonication procedure.^34^ Moreover, SUVs structure has a long circulation time and the passive target ability to diseased areas, while MLVs structure has an excellent encapsulation efficiency and slow release of lipophilic compounds.^38^ Therefore, most of current approved liposomal products are SUVs and MLVs.^39^

**TABLE 2:** Structural components present in liposomes and preparation method for liposomes.

Classification parameters	Comments/methods	Specifications	Reference
**Components for liposomes**			
Structural components	Phospholipids	Natural: cardiolipin PA: phosphatidic acid PC: phosphatidylcholine PE: phosphatidylethanolamine PG: phosphatidylglycerol PI: phosphatidylinositol PS: phosphatidylserine	^42^, ^43^, ^44^
		Synthetic: DOPE: 1,2‐dioleoyl‐sn‐glycero‐3‐phosphoethanolamine DLPC: 1,2‐dilauroyl‐sn‐glycero‐3‐phosphocholine DLPE: 1,2‐Dilauroyl‐sn‐glycero‐3‐phosphoethanolamine DLPG: 1,2‐Dilauroyl‐sn‐glycero‐3‐phosphoglycerol DMPC: dimyristoyl phosphatidylcholine DMPE: 1,2‐Dimyristoyl‐sn‐glycero‐3‐phosphoethanolamine DOPC: 1,2‐dioleoyl‐sn‐glycero‐3‐phosphocholine DPPC: dipalmitoyl phosphatidylcholine DPPG: 1,2‐dipalmitoyl‐sn‐glycero‐3‐phosphoglycerol DSPA: 1,2‐distearoyl‐sn‐glycero‐3‐phosphate DSPC: distearoylphosphatidylcholine DSPE: 1,2‐Dioleoyl‐sn‐glycero‐3‐phosphoethanolamine DSPG: 1,2‐distearoyl‐sn‐glycero‐3‐phospho‐(1’‐rac‐glycerol) DSPS: 1,2‐Distearoyl‐sn‐glycero‐3‐phospho‐l‐serine HSPC: hydrogenated soy phosphatidylcholine POPC: palmitoyl‐2‐oleoyl‐sn‐glycero‐3‐phosphocholine SLPC: palmitoyl‐2‐stearoyl(5‐DOXYL)‐sn‐glycero‐3‐phosphocholine	^45^, ^46^
	Sterol	Cholesterol Progesterone Saponins (ginsenoside Rh2, Rg3; holothurin A, echinoside A, and bivittoside D from sea cucumber, etc.) Phytosterols (β‐sitosterol, retinol, stigmasterol, etc.) Bacterial sterols (ergosterol, lanolin, etc.) Cholesterol derivatives (cholesterol hemisuccinate [CHEMS], lysine‐based cholesterol [CHLYS], cholesteryl arginine ethyl ester [CAE], etc.)	^47^, ^48^, ^49^, ^50^, ^51^, ^52^, ^53^, ^54^, ^55^, ^56^, ^57^, ^58^, ^59^, ^60^, ^61^
Additives	Hydrophilic polymers	PEG PEG derivatives, etc.	^20^, ^44^, ^62^, ^63^
	Ionic agents	Stearylamine (SA) Diethyl phosphate (DCP), etc.	
	Surfactants	sodium dodecyl sulfate (SDS) sodium octyl sulfate (SOS) Tween 80, etc.	
**Preparation methods for liposomes**			
Passive loading	Mechanical dispersion methods	The film hydration Microemulsification Ultrasonication method French pressure cell Membrane extrusion Dried reconstituted vesicles High‐pressure homogenization methods	^33^, ^64^
	Solvent dispersion method	Ethanol injection method Ether injection method De‐emulsification ‐Double‐emulsification ‐Reverse phase evaporation ‐Stable plurilamellar vesicles	^65^, ^66^, ^67^
	Detergent removal methods	Detergent dialysis Column chromatography Dilution Reconstituted Sendai virus enveloped vesicles	^68^
	Size transformation or fusion of vesicles	Dehydration–rehydration method Freeze‐drying (lyophilization) method	^69^, ^70^
Active loading	Suitable for certain compounds that are both water‐soluble and lipid‐soluble and have ionizable groups.		^71^, ^72^

The physicochemical properties and biological efficacy of liposomes can be influenced by various factors, including the type and size of liposomes, the ratio between different components, the surface charge of lipids, and modifications on the surface of particles.^40^ The surface charge of liposomes is typically determined by the hydrophilic polar head group of PLs. PLs, both natural and synthetic, are frequently utilized in the production of lipid‐NPs. Among these, phosphatidylcholine (PC) and phosphatidylethanolamine (PE) derived from plants and animals are the most commonly employed (Table 2).^39^ These PLs confer a negative (e.g., phosphatidic acid [PA], phosphatidylserine [PS], phosphatidylglycerol [PG], and cardiolipin), neutral (e.g., PC and PE) charges and positive (e.g. 3β‐[N‐(N′,N′‐dimethylaminoethane)‐carbamoyl]cholesterol (DC‐Chol), N‐[1‐(2,3‐dioleyloxy)propyl]‐N,N,N‐triethylammonium (DOTMA), 1,2‐dioleoyl‐3‐trimethylammoniopropane (DOTAP), 1,2‐dioleoyl‐ sn‐glycero‐3‐ethylphosphocholine (EDOPC), 2,3‐dioleyloxy‐N‐[2‐(sperminecarboxamido)ethyl]‐N,N‐dimethyl‐1‐propanaminium (DOSPA)) charge to liposomes at physiological pH.^32^, ^41^ Typically, liposomes or particles that are uncharged and possess low charge density exhibit favorable biocompatibility and stability. However, it is imperative to acknowledge the potential for side effects induced by their tendency to aggregate, which may lead to complications such as thrombosis. Section 2.3 provides a comprehensive discussion on cationic lipid (CLD)‐NPs.

### 2.2 Functionalized liposomes for cancer immunotherapy

As exogenous substances, liposomes may also interact with the immune system (e.g., progenitor cells, monocytes, and macrophages) and have been observed to accumulate to a greater extent in the liver and spleen, which are two major organs of the reticuloendothelial system (RES), compared with other organs. This accumulation has been linked to toxicity and depletion of immune cells, potentially leading to off‐target effects.^73^ Numerous approaches have been performed to optimize the formulation of conventional liposomes to reduce the off‐target toxicity, short circulation time, and in vivo instability (as depicted in Figure 3).^74^ Of all the approaches, the utilization of polyethylene glycol (PEG)ylated liposomes exhibits considerable potential to improve safety, allow for more efficacious immunotherapy, and is compatible with combination therapies. It has been found that PEGylated liposomes can significantly mitigate the effects of nonmodified liposomes on macrophages and liver function by reducing reticuloendothelial phagocytosis.^75^, ^76^ PEGylated liposomes that are covalently linked to immunostimulatory agents, specifically anti‐CD137 and interleukin (IL)‐2‐Fc, enables the retention of the robust antitumor effects of immune agonists or antibodies, while simultaneously mitigating the potential for heightened immune‐related adverse events.^77^, ^78^ This is because PEGylated liposomes create spatial barriers through the formation of “conformational clouds” and hydrated membranes on the liposome surface, thereby augmenting particle stability in circulation and improving EPR effect. This mechanism also prevents the clearance of liposomes by RES and facilitates targeting of the TME.^79^

Furthermore, as previously described liposomes can be modified with various targeting moieties to enhance their targeting properties.^32^ As an illustration, the internalization of liposomes coated with immunoglobulin M (IgM) by phagocytes was observed to be 100 times greater than that of uncoated liposomes or liposomes coated with unmodified IgM.^80^ Additional research group has documented that liposomes modified with a mesothelioma‐targeting human single chain antibody (M1) and radiolabeled with radioactive isotope indium‐111 exhibited a prolonged half‐life in plasma and a significant accumulation in the tumor region 48 h after intravenous administration, in contrast to the minimal uptake observed in tumors treated with untargeted liposomes. Notably, this liposome formulation also demonstrated the ability to contribute to early diagnosis through single photon emission computed tomography (SPECT/CT) imaging.^81^ Guo et al.^82^ successfully developed liposomes that carry anti‐CD44 antibodies and anti‐IL6R antibodies that specifically target the TME of CD44^+^ breast cancer cells, thereby inhibiting metastasis in breast cancer mouse models.

Stimulus‐responsive liposomes hold great potential for optimizing the effectiveness of conventional liposomal drug delivery. Through the manipulation of lipid ratios with specific stimulus‐response mechanisms such as changes in temperature or pH and in response to exposure to specific enzymes (e.g., matrix metalloproteinases [MMPs], prostate‐specific antigenases, cathepsin, elastases, hyaluronidase, and Urokinase‐type plasminogen activator, etc.). Therefore, liposomes are enabled to provide heightened selectivity and controlled release of drugs during treatment, leading to decreased systemic toxicity, improved physicochemical properties, and enhanced antitumor efficacy.^83^, ^84^ A pH‐sensitive liposomal vaccine was synthesized successfully by Yuba et al.^85^ through modifying pH‐sensitive dextran derivatives onto the surface of ovalbumin (OVA) molecules‐loaded liposomes. The resultant liposomal vaccine demonstrated stability at physiological pH, but was found to be destructible in the tumor region which has a weakly acidic pH. Further study revealed that the pH‐sensitive OVA vaccine was capable of inducing more effective antigen‐specific immunity and tumor inhibitory efficiency as compared with the unmodified liposomes loaded with OVA.^85^, ^86^ Moreover, enzyme‐responsive liposomes are an important part of stimulus‐responsive liposomes, which are characterized by triggering drug release via attaching substrates of tumor‐specific enzymes at the response site.^87^ In a recent study, a pH and MMPs dual‐responsive liposome was developed by the surface modification of pH‐responsive polymer‐PEG2000 and MMP‐responsive peptide (GPLGVRG), that conjugated with a programmed death‐ligand 1 (PD‐L1) antagonist (hydrolysis resistant D‐peptide NYSKPTDRQYHF). These dual‐responsive liposomes enable improved biodistribution and on‐demand release of immune checkpoint inhibitor (ICI) molecules within tumor regions via specific cleavage of tumor regionally secreted MMP‐2 enzymes following injection. Simultaneously, the accelerated release of doxorubicin (DOX) was observed in the acidic TME.^88^

Overall, the unique size and properties of liposomal NPs provide numerous advantages, particularly improved biocompatibility and biodegradability, and increased safety when compared with polymeric and inorganic NPs. Additionally, the lipid/oil solubilizing capacity of these liposomes enhances the solubility of hydrophobic agents. Furthermore, liposomes have the ability to improve bioavailability through the prevention of agent degradation and enhancement of gastro‐intestinal membrane permeability, enhance targetability and controlled drug release. Moreover, multifunctional liposomes also offer apromising and low‐toxicity alternative for a diverse array of drugs that were previously restricted by low therapeutic index or undruggable characteristics. It is reasonable to assert that these liposomes, which possess specific targeting, stimulus‐responsive, combined therapy, and imaging capabilities, will play a pivotal role in significant advancements toward improving the safety and therapeutic benefits for individuals with cancer.^89^

### 2.3 Delivery of therapeutic nucleic acids system by cationic LNPs

Novel RNA‐based immunomodulators, such as mRNA vaccines, antisense, small interfering RNA (siRNA), and mRNA‐based immune drugs, have been developed.^90^ Among these, mRNA therapeutics have gained significant attention in cancer immunotherapy due to their ability to induce robust long‐term immune responses. The Pfizer‐BioNTech (BNT162b2) and Moderna (mRNA‐1273) COVID‐19 liposomal vaccines, which utilize mRNA encoding the SARS‐CoV‐2 spike protein as antigens to elicit host immune responses, represent the most compelling successes in this field.^91^, ^92^, ^93^ These vaccines are PEGylated cationic LNPs with a diameter of 80–100 nm. The CLDs used in the BNT162b2 and mRNA‐1273 vaccines are tertiary amines, specifically ALC‐0315 (Pfizer) and SM‐102 (Moderna), respectively.^94^, ^95^ These vaccines have demonstrated successful efficacy in delivering SARS‐CoV‐2 mRNA for the prevention of COVID‐19 and indicating the huge promise of LNPs as suitable carriers for emerging RNA‐based immunotherapies.^90^ Specifically, cationic LNPs have facilitated the feasibility and efficiency of mRNA‐based immunotherapy strategies by safeguarding and delivering new immunotherapy‐related RNA constructs (such as self‐amplifying RNA and circular RNA, microRNA [miRNA] and siRNA for the modification of translation, as well as CRISPR associated protein 9 messenger RNA [Cas9 mRNA] and single guide RNA [sgRNA] used for gene editing) to specific organs or cell types, thereby stimulating the immune response.^96^

Cationic LNPs are defined as structures resembling liposomes, with an average size ranging from 80–100 nm, and consisting of four primary constituents: (1) PLs for drug encapsulation, (2) cholesterol for stability maintenance, (3) cationic or ionizable lipids that interact with polyanionic RNA to enhance drug‐loading capacity and facilitate endosomal escape, and (4) lipid‐anchored PEG for improved circulation and reduced immune system recognition.^97^ The ionizable CLDs in LNPs are neutrally charged at a physiological pH of 7.4 to minimize the likelihood of aggregation with blood components, while exhibiting a positive charge under acidic conditions (pH∼4) to promote RNA complexation.^98^ CLDs have the ability to establish stable complexes with negatively charged mRNA through electrostatic attractions, thereby impeding the binding of mRNA with serum albumin, safeguarding them from degradation by endogenous nucleases, and evading clearance by the host immune system.^99^ A considerable synthetic endeavor has led to the discovery of numerous CLDs, including multivalent CLD, multitailed ionizable PLs (iPhos), DC‐chol, DOTMA, dimethyldioctadecylammonium, DOTAP, ethyl PC's, GL67, and DODMA.^100^ These CLDs exhibit structural similarities to their natural counterparts, typically comprising a hydrophobic lipid tail and ionizable (cationic) head groups that contain amine moieties, amino moieties, or choline moieties. The caveat here is that the efficacy of permanently charged quaternary amine moieties in the hydrophilic head group region for siRNA delivery has been reported to be inferior to that of other lipids.^101^ Further investigation has revealed that ionizable amino lipids with an apparent acid dissociation constant value (p*K*a) between 6.2 and 6.5 are effective for delivering therapeutic siRNAs.^102^ For optimal therapy the goal of the mRNA–lipid complexes is to reach specific target cells and encode sufficient proteins of interest. Associated to this, cationic LNPs tend to bind to negatively charged molecules of the cell membrane via electrostatic interaction, resulting in enhanced permeability and intracellular uptake.^103^ Upon internalization, these structural LNPs facilitate efficient delivery of the payload by enhancing intracellular phagocytosis through complexation with the negatively charged endo/lysosomal membrane. This, in turn, accelerates drug release and enables the exertion of its effect in the cytosol.^104^

Recent studies have investigated the feasibility of LNPs for packaging mRNA products (e.g., single‐stranded mRNA or engineered circular RNA encoding TAA) as unique cancer neoantigens, for presentation on major histocompatibility complex (MHC) to APCs.^105^, ^106^, ^107^ Zhang et al.^108^ revealed that LNPs improve OVA expression in DCs by increased intracellular delivery of OVA‐mRNA, achieving significant beneficial effects in tumor prevention and therapeutic antitumor efficacy. Importantly, a specific 12‐carbon tail lipid endowed this LNP with adjuvant properties through Toll‐like receptors (TLRs)‐4 signaling activation further enhanced the antitumor function of a mRNA vaccine. Additionally, it was established that LNPs can be utilized to load mRNA to enhance the production of immunomodulatory proteins, such as TLR, costimulatory ligands on T cells or B cells, chemokines, and different mAb formats in various cell subpopulations.^45^ As an illustration of the latter, intratumoral administration of LNPs, which have an average size of 150–200 nm and contain mRNA encoding IL‐12, IL‐27, and GM‐CSF, were found to elicit anticancer effects. In the mouse B16F10 melanoma xenograft model, repetitive administration of DAL4‐LNP resulted in effective inhibition of tumor growth by inducing adequate infiltration of immune effectors into the tumor without causing systemic toxicity.^109^ A separate investigation revealed that the novel DAL4‐LNP, comprising DOPE, cholesterol, PEG, di‐amino lipid materials (DALs), and mRNA, demonstrated the ability of LNPs to safeguard mRNA sequences for effective in vivo administration, leading to significant production of anti‐human epidermal growth factor receptor 2 (HER2) antibodies in serum for a duration of 14 days (45 ± 8.6 μg/mL) following LNPs injection in breast cancer mice.^110^ The exploration of LNPs, which carry double‐stranded siRNA for the purpose of knocking down checkpoint inhibitors such as anti‐PD‐L1, indoleamine 2,3‐dioxygenase 1 (IDO), and transcription factor forkhead box P3 (FOXP3), among others, is being investigated for the treatment of cancer, as well as for in vitro engineered DC vaccines.^111^ Moreover, the utilization of LNPs for the codelivery of antigen‐encoding‐ and immunomodulatory‐encoding‐mRNA has demonstrated the ability to activate innate immunity through the binding of PRRs expressed by APCs.^112^, ^113^, ^114^ Additionally, the codelivery of immunomodulatory‐encoding mRNA with siRNA for checkpoint inhibitors was shown to counteract the immunosuppressive TME and achieve potent antitumor efficacy.^115^, ^116^  Advancements in LNP technology, which incorporate CRISPR complexes, have demonstrated their potential as adaptable formulation platforms for the delivery of chimeric antigen receptor (CAR), T cell receptor, and/or circular RNA into lymphocytes, thereby facilitating CAR‐T therapy.^117^, ^118^, ^119^, ^120^, ^121^, ^122^, ^123^, ^124^, ^125^, ^126^ Furthermore, recent research has produced multiplexed dendrimer LNPs capable of codelivering siRNA, Cas9 mRNA, and sgRNA, indicating its potential for precise delivery and improved gene‐editing effectiveness.^127^, ^128^, ^129^

While LNPs represent a significant breakthrough for mRNA delivery into cells, it is crucial to consider the potential benefits of targeted delivery of mRNA‐LNP vaccines to lymph nodes (LNs) in terms of improving immune responses and mitigating adverse effects such as hepatic damage.^130^ Therefore, the targeted expression of mRNA in specific organs and cells in vivo is widely regarded as the primary challenge for cationic LNPs‐mediated mRNA delivery. To address this challenge, optional bio‐targeting layers, stimulus‐responsive motifs based on temperature, pH, or magnetic fields, and controllable component types could be employed to enable the systematic and accurate therapy or gene editing.^117^, ^123^, ^124^, ^125^, ^131^ Sakurai et al.^132^ reported the accumulation of RGD‐modified LNPs in the vasculature of metastasized cells in a murine model of breast cancer with lung metastasis, but not in healthy cells. It is noteworthy that modifying the structural components, and incorporating supplementary compositions also evince organ or cell targeting to some extent.^118^, ^133^, ^134^, ^135^, ^136^ In comparison with smaller LNPs that possess a consistent formulation, larger particles containing siRNA or mRNA (>200 nm) exhibited increased cellular uptake in splenic DCs via micropinocytosis.^137^ Furthermore, the incorporation of negatively charged 1,2‐dioleoyl‐sn‐glycero‐3‐phosphate (18PA) can function similarly to DOTAP in conferring organ specificity.^118^ The ongoing progress in technology such as DNA/RNA barcode or in combination with flow cytometry for the identification of ionizable lipid targeted various organs/tissues/cells suggests significant promise for LNPs‐based mRNA therapeutic applications.^138^ We anticipate that targeted LNPs will be developed in the near future to achieve heightened immune responses at reduced RNA dosages.

### 2.4 EVs: Attractive candidate platforms for next‐generation immune drugs delivery

EVs, including exosomes, microvesicles, and so on, which are PL nanovesicles (NVs), are secreted extracellularly by a wide range of mammalian cell types, including animal cells, plant cells, and microorganisms. Naïve EVs are comprised of cell membranes that bear numerous adhesion proteins on their surface, exhibit multivalency, and possess the ability to target cells. Their inherent physicochemical properties, including appropriate size, hydrophilic shell, low aggregation potential, and cell‐derived antiphagocytic markers, facilitate evasion of RES clearance.^139^ The diameter size of EVs typically ranges from 30 to 150 nm, depending on their origin and function.^140^ EVs are enriched with various biomolecules, including proteins, lipids, enzymes, transcription factors, and nucleic acids such as DNA and RNA (mRNAs, miRNAs, and long noncoding RNAs [lncRNAs]).^141^ They are capable of encapsulating and delivering functional molecules to recipient cells or tissues via body fluids.^142^, ^143^ Research has substantiated that cancer cells secrete a greater quantity of EVs as compared with nonmalignant cells (e.g. immune cells and epithelial cells). These tumor‐derived EVs (TDEVs) contain molecular components specific to tumors and can be extracted from blood and urine. Considering the important role of TDEVs in the progression of cancer from early stages to metastasis, they have the potential to serve as the next generation of fluid biopsy biomarkers for cancer diagnosis and management.^144^, ^145^ Furthermore, TDEVs with targeting abilities to transfer functional proteins or nucleic acids to selective cells have demonstrated inspiring preclinical outcomes in cancer nanomedicine and immunotherapy.^146^, ^147^ But it should be noted that TDEVs as a drug carrier should be engineered by changing the original cargos into therapeutic products or altering modification on the surface of the membrane, thereby preventing the tumor‐promoting effect of TDEVs from counteracting the anticancer efficacy (engineered EVs will be discussed subsequently).^148^

Furthermore, the self‐immunomodulatory attribute of exosomes facilitates the progression of novel immunological methodologies in the field of oncology, by activating adaptive and innate effector cell‐mediated immune surveillance.^146^, ^149^ Certain exosome variants retain the crucial immunostimulatory characteristics of their progenitor cells and can function as cancer vaccines, augmenting the scope and potency of the immune response.^150^ For instance, TDEVs enriched with TAA can elicit cross‐priming of cytotoxic T lymphocytes (CTLs).^151^ DC‐secreted exosomes possess MHC class I or MHC class II peptide complexes, costimulatory molecules (e.g., CD40, CD80, and CD86), tumor necrosis factor (TNF) superfamily ligands (e.g., TNF, FasL, and TRAIL), and NK group 2, member D (NKG2D) ligands (e.g., UL16 binding protein 1) that facilitate antigen presentation and promote the activation of tumor‐specific CTLs.^152^, ^153^, ^154^ Furthermore, exosomes derived from other immune subpopulations may also possess the capacity to elicit effective antitumor immunity for cancer immunotherapy.^155^ An illustration of this phenomenon is the secretion of exosomes by B cells, which contain MHC‐II‐like peptide complexes that stimulate the clonal expansion of CD4^+^ T cells and modulate cytokine secretion.^156^ Additionally, macrophages carrying pathogen‐associated antigens promote dendritic cell maturation and the release of proinflammatory cytokines upon pathogen exposure. M1 macrophages have been observed to release exosomes that effectively reprogram immunosuppressive M2 macrophages to immunoresponsive M1 macrophages.^157^ The immunomodulatory capabilities of EVs can be enhanced by directly encapsulating biological cargos such as antigens, immune adjuvants, immune checkpoint molecules, and so on.^146^ Generally used immune adjuvants for promoting DC maturation are cytokines, cytosine‐phosphorothioate‐guanine oligonucleotides (CPG ODN), TLR agonists, STING agonists, and LPS derivatives, and so on.^158^ Adamus et al.^159^ employed a method of encapsulating CpG‐STAT3 antisense oligonucleotide conjugates into EVs derived from neural stem cells (NSC), which resulted in a significant enhancement of the immunoreactivity of natural NSC‐derived exosomes. This approach effectively targeted glioma‐associated microglia and inhibited tumor growth.^159^ Furthermore, EVs demonstrated the potential to amplify the antitumor immune effects induced by conventional ablative therapies, such as low‐dose chemotherapy, radiation therapy, and PDT, without causing intolerable systemic toxicity.^160^ EVs, therefore, offer a rational platform for the combined administration of ablative therapies. Wang et al.^161^ conducted a study wherein EVs were modified to simultaneously transport the secondary generation photosensitizer chlorin e6 (Ce6) for PDT and the TLR agonist R848 for the treatment of prostate cancer. The utilization of EVs in this context effectively addresses the prevailing issues associated with photosensitizers, such as nonspecific accumulation and inadequate water solubility, while also improving antitumor efficacy through the induction of cascade immune responses.^161^

In addition, on the basis of directly incorporating biological cargos into EVs, engineered EVs have been designed to further improve targeting, optimize biological distribution, and enhance the immunogenicity and antitumor efficiency of immune drugs.^162^, ^163^, ^164^ Common methods are endogenous engineering of donor cells with homing peptides and appropriately decorating the surface of EVs with antigen‐associated signaling molecules, aptamers, and small molecular drugs.^146^, ^165^ For example, the cDNA encoding the target OVA homing peptide was inserted into the gene sequence of donor EG7 tumor cells by genetic engineering techniques (transfection), thereby altering the structure of the secreted exosomes to express OVA on their cell membranes. Moreover, the OVA‐pulsed DC‐derived EVs exhibit a stronger stimulatory effect than engineered TDEVs in the induction of OVA‐specific CD8^+^ T cell responses and antitumor immunity, and both of them showed tumor targeting ability.^166^ A variety of transmembrane proteins have been tried to build engineered EVs with homing ability, such as gap junction protein connexin43, lysosome‐associated membrane protein‐2b, fibronectin/tenascin‐C, CD protein (LA), and four transmembrane proteins (CD63, CD9, and CD81).^167^, ^168^, ^169^ Mesenchymal stem cells (MSCs)‐derived exosomes were found to be minimally immunogenic and immunity‐inducible, as well as suitable for low‐cost mass production.^170^ In a recent study, a codelivered biological platform was developed that is based on bone marrow MSCs that electroporation‐loaded galectin‐9 siRNA and modified with surface‐bound oxaliplatin prodrugs for the enhanced treatment of pancreatic cancer. The engineering EVs have been found to be effective in inducing immunogenic cell death (ICD), and providing sufficient antigen for antitumor immune responses. Moreover, the EVs have the ability to modulate tumor suppressor macrophage polarization and reduce Tregs' levels, thereby remodeling the TME.^171^ Engineered exosomes based on macrophages are effective strategy to modulate the TME in cancer immunotherapy.^172^ Gunassekaran et al.^173^ extracted exosomes from M1 macrophages, which were subsequently engineered to load nuclear factor kappa‐light‐chain‐enhancer of activated B (NF‐κB) p50 siRNA plus miR‐511‐3p and target the IL4 receptor, named IL4R‐Exo (si/mi). Systemic administration of IL4R‐Exo (si/mi) showed effective tumor targeting by re‐educating TAM into M1‐type macrophages and promoting the activity of immunostimulatory cells to remodel TME, resulting in inhibited tumor growth.^173^

Moreover, the biocompatibility, tumor targeting, and controlled release of EVs can be substantially enhanced by physically modifying their surface with liposolubility targeting molecules or PEG coatings.^174^, ^175^ One example of this is the creation of a genetically engineered multifunctional immunomodulatory exosome (GEMI‐NI‐Exos), which utilizes Expi293F cell‐derived EVs that are encapsulated with PD‐1 and OX40 mAb, and surface‐modified to target CD3 and epidermal growth factor receptor (EGFR). GEMINI‐Exos effectively targets EGFR‐positive breast cancer cells, and its injection induces robust anticancer immunity and efficient tumor suppression in a mouse model of breast cancer.^176^ It is noteworthy that PEG coatings may potentially conceal other inherent bioactive surface functionalities of EVs. Furthermore, modification of the molecular structure of EVs film by click chemical method is another engineering strategy.^177^ Koh et al.^178^ developed a pH‐sensitive engineered exosome by utilizing a dibenzocyclooctyne‐modified antibody to CD47 and SIRPα (aCD47 and aSIRPα) to bind azide‐modified EVs of M1 macrophages. The engineered EVs demonstrated superior RES camouflage, prolonged circulation half‐life, and regulated release, thereby exhibiting the potential for reversing tumor immunosuppressive TME. Additionally, EVs are used to prepare exosomal membrane‐coated NP particles such as PLGA NPs to reduce the immune clearance of NPs and improve their tumor‐specific targeting.^179^ Furthermore, membrane fusion methods can be chosen for those EVs which are difficult to modify ligands directly on EVs membranes, also in case for hybrid EVs preparation in which lipids (DOTAP, POPC, DPPC, and POPG) are fused with EVs.^180^, ^181^, ^182^ As such, the outlook on the future of EVs as delivery approach for cancer immunotherapy is promising but researchers should consider that the intricate composition of engineered immune EVs during nanomedicine design phase may pose challenges in their clinical implementation and large‐scale manufacturing.

### 2.5 SLNs and NLC for the large‐scale production

The emergence of nanotechnology has led to the development of SLNs as a new generation of colloidal NPs, ranging in size from 10 to 1000 nm.^183^ Unlike conventional liposomes that contain liquid crystal lipid bilayers, SLNs possess a micelle‐like structure composed of natural or synthetic solid lipids, surfactants, and other coating materials.^184^ Common solid lipids include triglycerides, fatty acids, steroids, and waxes, which can solubilize hydrophobic drugs in their melted lipid phase or form a drug‐enriched shell surrounding them.^185^ Surfactants are chemical compounds composed of a polar (hydrophilic) head and a nonpolar (hydrophobic/lipophilic) tail. The incorporation of surfactants facilitates the stabilization of SLNs by reducing the interfacial tension (adhesive forces) between two phases.^186^ It has been observed that SLNs exhibit superior efficacy in encapsulating hydrophobic drugs in comparison with liposomes.^187^ Additionally, SLNs offer greater control over drug release in comparison with other types of lipid‐NPs. The encapsulation of drugs within SLNs can be achieved through physical adsorption, adsorbents, and coprecipitation, while the modulation and optimization of SLNs release are through adjusting formulation into a drug‐enriched core or shell pattern.^188^ This is mediated by the rapid diffusion of drugs adsorbed on the surface of SLNs during the initial release phase, while drugs encapsulated in the interior of SLNs exhibit a prolonged release profile due to nonuniform distribution within the lipid matrix.^189^

Various production methods, including high‐pressure homogenization, ultrasonic emulsification, thermal melting, and fatty acid emulsification, have been employed to prepare SLNs without the use of organic solvents.^189^ The inclusion of solid lipids in SLNs confers superior physical and chemical stability compared with liposomal and polymeric NPs, thereby enabling the production of sterile products. Additionally, these characteristics contribute to a reduction in lipid degradation, which renders SLNs suitable for large‐scale production and reproducibility.^190^ The rather straightforward manufacturing process and adaptable carrier properties, including enhanced drug‐loading capacity, improved protein stability, encapsulation efficiency, and precise control, further enhance the clinical potential of SLNs for localized and systemic cancer applications.^191^, ^192^, ^193^, ^194^, ^195^ Banerjee et al.^196^ presented a paclitaxel formulation based on SLNs that was engineered with Tyr‐3‐octreotide (PSM) and demonstrated the ability to induce ICD. This formulation was found to modulate local CD8^+^ T cell infiltration and systemic immune responses in melanoma mice following administration.^196^ Despite these promising results, further research is needed to investigate the incorporation of clinical immunotherapy cargoes into SLNs for cancer treatment.

Upon storage, the solid lipids present in SLNs have a tendency to undergo crystallization, leading to the expulsion of the encapsulated drug into the surrounding medium. This, in turn, results in a decrease in the encapsulation efficiency of SLNs.^186^, ^197^ To overcome these limitations of SLNs, NLCs have been developed. NLCs are designed to enhance drug loading, prevent drug leakage, and achieve controlled drug release under certain conditions.^198^ Unlike SLNs, NLCs are capable of inhibiting drug release during storage by incorporating a small amount of liquid lipids into the solid lipids. This reduces the crystallinity of the lipid core and improves the overall stability of the formulation.^199^ Furthermore, the incorporation of liquid lipids has the potential to disturb the lattice arrangement of solid lipids, leading to the formation of anomalous crystalline cores within NLCs, thereby augmenting the drug loading capacity by increasing the available space.^199^, ^200^ By manipulating the proportion of liquid lipids while preserving the skeletal structure of NLCs can facilitate more accurate regulation of drug release kinetics.^201^ Although the field is still young, the deployment of SLNs and NLCs encapsulating widely used cancer immune drugs and some mRNA products hold an encouraging potential for future studies.

### 2.6 Nanoemulsions

NEs are characterized as biphasic dispersions consisting of two immiscible liquids, namely, a dispersed phase and a continuous phase (water and oil), which are typically stabilized through the use of surfactants and cosurfactants as emulsifiers.^202^ The formation of NE is not typically spontaneous, but rather requires the application of energy input through high pressure homogenizers, high shear agitation, or ultrasonic generators.^202^ The prevalent forms of emulsions comprise a “water‐in‐oil” (w/o) emulsion, an “oil‐in‐water” (o/w) emulsion, and multiple emulsions, which are contingent upon the proportion of water to oil and the type of emulsifier employed.^203^ For instance, emulsifiers that are more soluble in water generally produce w/o emulsions, with their nonpolar tails extending into the oil and their polar head groups oriented toward the water. Conversely, emulsifiers that are more soluble in oil generate o/w emulsions, with their polar heads extending into the water and their nonpolar tails directed toward the oil.^204^ The physiochemistry of NE, specifically their drug loading capability and drug release rate, has been found to be dependent on several factors including the water content, types and contents of oils/lipids (typically 5–20 wt% of the NE), and the oil/water partition coefficient of the drug molecule in the NE system. Commonly utilized oils/lipids for NEs consist of long‐chain unsaturated fatty acids, glycerides, vegetable oils, medium‐chain triglycerides, and polyalcohol esters of medium‐chain fatty acids.^205^ Consequently, the screening of oils/lipids has been demonstrated to be a crucial aspect of NE development, and novel oils and lipids are currently being developed for use in synthetic NE. One such example is deep‐sea fish oil, which has been demonstrated to be a safer therapeutic option for bleomycin‐induced pulmonary fibrosis in BALB/c mice.^206^, ^207^ NE exhibit excellent kinetic stability, and the addition of appropriate surfactants can further enhance their stability by reducing the oil–water interfacial area and tension, as well as through electrostatic interactions with the interfacial surface.^208^ For instance, oil‐in‐water emulsions containing a soy protein/soy polysaccharide complex as surfactants have been shown to maintain stable oil droplet size.^209^

NEs are significant contributors to various diverse fields, including drug carriers, food, cosmetics, and oilfield chemicals.^210^ In the context of drug delivery, NEs have exhibited the capacity to efficiently load lipophilic drugs and nutrients, such as fats, carbohydrates, and vitamins, into oil droplets. This process enhances their dispersion and stability in aqueous solutions and facilitates drug release and absorption postdigestion.^207^ Furthermore, due to their small droplet size, NEs are considered one of the most promising options for oral drug delivery.^211^ The current state of research regarding the use of NEs to facilitate immunotherapy is in its preliminary stages, with a primary focus on the administration of chemotherapeutic drugs such as tamoxifen and dacarbazine, as well as tumor multimodal imaging.^212^, ^213^, ^214^, ^215^, ^216^ However, initial findings suggest that NEs may hold significant potential in the realm of cancer immunotherapy. Specifically, a tailored NE formulation containing perfluorooctyl bromide and MnO_2_ NPs has been developed as a theragnostic agent, capable of providing both magnetic resonance imaging (MRI) and computed tomography (CT) dual‐modality imaging, as well as inducing advanced ICD.^217^ Furthermore, Zeng et al.^218^ reported data indicating the efficacy of a novel NEs platform in promoting the targeting of DCs with TAA or neoepitopes, thereby inducing tumor‐specific immunity with therapeutic potential. Current endeavors aim to explore the potential of NEs in the context of cancer immunotherapy.

### 2.7 The upgraded version of lipid‐NPs: LPHNPs

The application of lipid‐NPs and polymeric NPs has surfaced as efficacious methodologies to enhance the solubility and stability of anticancer drugs, protract the half‐life of anticancer drugs in plasma, diminish off‐target distribution, mitigate drug toxicity, and foster drug accumulation in disease regions, thereby augmenting the therapeutic efficacy of the loaded drugs.^219^ Nevertheless, both liposomal and polymeric NPs exhibit certain limitations, prompting the development of LPHNPs as a novel class of NPs.^220^

LPHNPs can be classified into five distinct classes based on their structural composition, including monolithic LPHNPs, polymer core lipid shell NPs, hollow core lipid–polymer–lipid nanoparticles, biomimetic lipid polymer hybrid systems, and polymer caged liposomes.^221^ Despite their subtype, LPHNPs offer several advantages, such as enhanced biocompatibility and safety, improved drug loading capacity and controlled release, as well as prolonged circulation time and therapeutic efficacy of drugs.^222^ For example, the utilization of CLD‐based cores has demonstrated the ability to incorporate anionic drugs and nucleic acids.^223^ This approach can be further enhanced by utilizing lipids or biomimetic lipids, such as those derived from stem cells, platelets, and white blood cells, as a coating layer. This coating layer can reduce the potential for unexpected immune reactions and toxicity, improve biocompatibility, prevent drug excretion, and delay drug release.^224^, ^225^ Additionally, the incorporation of amphiphilic lipids as a lipid coat can aid in stabilizing the matrix.^226^ Furthermore, the incorporation of a polymeric core, such as a PLGA core, in LPHNPs can facilitate the incorporation of both hydrophilic and hydrophobic drugs, preventing their systemic metabolism, and promoting enhanced storage stability.^227^ In comparison with SLNs and liposomes, LPHNPs exhibit greater suitability for hydrophilic drugs due to their higher drug encapsulation rate. This is attributed to the electrostatic adsorption effects that enable the formation of drug–polymer complexes, which are subsequently encapsulated in hydrophobic lipids.^227^, ^228^ Notably, the presence of a PEG layer on the lipid core of LPHNPs confers highly adjustable characteristics and improved physicochemical stability.^229^ That is becausethe integration of the lipid–PEG layer into the lipid shell of LPHNPs imparts surface hydrophilicity, prevents absorption by the RES, prolongs circulation time, and improves its steric stability in plasma matrices.^230^ Also, this approach has demonstrated success in enabling the traversing of various physiological barriers.^231^

In particular, numerous immune agents have undergone testing for delivery efficiency and antitumor therapeutic index by LPHNPs, including refractory chemotherapy drugs, targeted drugs, and mRNA.^232^, ^233^ One example involves the encapsulation of OVA‐encoding mRNA into hybrid core–shell NPs to create a nanovaccine. This nanovaccine has been shown to enhance vaccination effects specifically in DCs due to macropinocytosis, resulting in improved antitumor growth efficiency and reduced metastases in the lungs of B16‐OVA‐bearing mice following repeated injection.^234^ The introduction of additional immune activators, such as various TLR agonists, along with antigens into LPHNPs, has the potential to enhance antigen uptake, improve lysosome escape, increase cytokine production, produce depot effects, and promote trafficking to secondary lymphoid organs. Consequently, these DC‐targeted vaccines can elicit potent and long‐lasting immunity against cancer.^235^, ^236^ The introduction of additional immune activators, such as various TLR agonists, along with antigens into LPHNPs, has the potential to enhance antigen uptake, improve lysosome escape, increase cytokine production, produce depot effects, and promote trafficking to secondary lymphoid organs. Consequently, these DC‐targeted vaccines can elicit potent and long‐lasting immunity against cancer.^237^ These hybridized particles are being considered as a promising platform for adapting combined anticancer modalities that work through boosting host immunity synergistically. For example, various approaches such as image‐guided cancer immunotherapy, chemoimmunotherapy (e.g., CD47 siRNA + etoposide), photoimmunotherapy (e.g., indocyanine green + immune stimulatory recombinant protein FimH; IR‐780 and imatinib; pyropheophorbide‐lipid conjugate [pyrolipid] + oxaliplatin; and recombinant PD‐1 decorated thermal responsive LPHNPs, etc.), multitherapeutic strategy (e.g., Prominin2 siRNA + oxaliplatin + ferroptosis) have been developed.^238^, ^239^, ^240^, ^241^, ^242^, ^243^ In general, LPHNPs, an advanced form of SLP, represent a promising type of nanomaterial with extensive possibilities and potential applications in the areas of RNA therapeutics, targeted immunotherapy, combination immunotherapy, and as an all‐in‐one platform for diagnosis and treatment.

## 3 cGAS–STING, A PIVOTAL PATHWAY IN CANCER IMMUNOTHERAPY

Under immunosurveillance, the evolution of malignant cells gives rise to tumors capable of evading immune detection or suppressing immune attacks.^244^ Immuno‐oncology therapy aims to overcome immunosuppression and reinvigorate the antitumor immunity of cancer patients by blocking immunosuppressive pathways in TME or activating the antitumor immune signals. Cancer immunotherapy has developed rapidly in the past decade, in particular through the success of immune checkpoint‐related therapies.^245^ Antibodies that target PD‐1, PD‐L1, lymphocyte‐activation gene 3 (LAG‐3), and CTLA‐4 have been approved by US FDA for the treatment of various solid or advanced tumors and have shown unprecedented efficacy and long‐lasting response. However, clinical data indicate that sole treatment with ICIs to disarm negative signals is ineffective in those cancer patients without sufficient antitumor immunity.^246^

**FIGURE 4:**
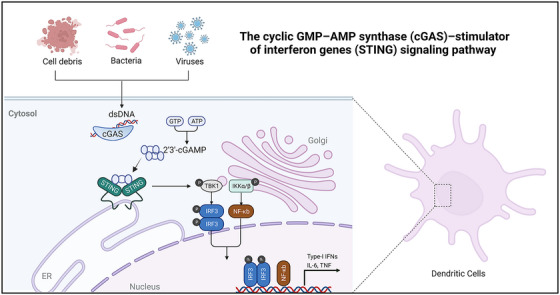
Overview of the cyclic GMP–AMP synthase (cGAS)–stimulator of interferon genes (STING) signaling pathway in dendritic cells (DCs). Figure was created using biorender.com.

Through the in‐depth exploration of tumor immune activation‐related pathways, an increasing number of potentially therapeutic targets have been identified, including the cyclic guanosine monophosphate (GMP)–adenosine monophosphate (AMP) synthase (cGAS)–STING signaling pathway, a widely recognized endogenous sensor of tumors. cGAS is an enzyme that was originally discovered as a cytoplasmic PRR for the detection of pathogen DNA. It can be initiated directly by cytosolic double‐stranded (ds)DNA from invading microbes such as DNA viruses, retroviruses, and intracellular bacteria (Figure 4).^247^ In brief, dsDNA enters the cytosol and induces cGAS activation by directly binding to the endoplasmic reticulum (ER). This activated cGAS generates cyclic GMP–AMP (cGAMP), a negatively charged CDN complex in the presence of guanosine‐5'‐triphosphate and adenosine triphosphate. cGAMP functions as a second messenger that binds to the adaptor protein STING in the ER and undergoes dimerization, in turn translocating to the Golgi apparatus and triggering activation of TANK‐binding kinase 1 (TBK1)/IFN regulatory factor 3 (IRF3) signaling.^248^, ^249^ Alternatively, STING in the form of a dimer can also activate the NF‐κB signaling via IκB kinase‐mediated phosphorylation of IκBα.^248^ Thus, STING pathway activation leads to the transcription of type I IFN‐associated genes and genes encoding proinflammatory cytokines (e.g., TNF and IL‐6), which promotes antitumor immunity through the activation of T cells and NK cells.^250^, ^251^, ^252^ Although initially discovered as a PRR for DNA from invading pathogens, under some conditions, cGAS–STING pathway can also be activated by self‐DNA leading to autoimmune disorders.^253^ In tumors, DNA or cGAMP from dying tumor cells access the cytosol of APCs and activate the cGAS–STING pathway, leading to the induction of type I IFNs and other immune stimulatory molecules, which are essential in modulating the functions of immune cells.^254^ Moreover, cancer treatments that include radiotherapy and chemotherapy which induce DNA damage and genomic instability also showed the ability to activate the cGAS–STING pathway and the development of antitumor immune responses.^255^, ^256^ The biological effects of STING activation on antitumor immunity will be discussed detail in the next section.

Given the importance of the cGAS–STING pathway in cancer immunity, considerable effort has been put into targeting cGAS–STING pathway pharmacologically. Numerous agonists for cGAS–STING pathway have been developed and shown effective antitumor effects. In order to upregulate STING expression, STING agonists must penetrate into cells to reach their target. Several CDNs from cGAMP and non‐nucleoside small molecule STING agonists are in different stages of clinical development, which will be covered in detail in Section 4.1. However, one of the major concerns in the clinical usage of STING agonists is that naked STING agonists are poorly internalized by cells. Moreover, potential adverse effects associated with cytokine induction also constrain the application of STING agonists. The development of STING agonists has entered a bottleneck as most novel‐immunodrugs. Therefore, the current investigation focused on delivering STING agonists with diverse vectors. These vectors can be cationic LNPs, liposomes, polymeric NPs and metal NPs. which are the most commonly used carrier system. Among these biomaterials, the lipid‐NPs have advantages of good biocompatibility and safety, low immunogenicity, and biodegradability as described previously. Considering that STING agonists cover the advantages and challenges of the most immune activation adjuvants, and STING agonists are taken as a representative in the subsequent part of this review to discuss the potential of lipid‐NPs application in cancer immunotherapy. The aim of lipid‐NPs for STING agonists is to improve safety and enhance intracellular delivery efficacy, to thereby exploit the full therapeutic potential of STING agonists in immunotherapy.

### 3.1 Role of cGAS–STING pathway on tumor cells

**FIGURE 5:**
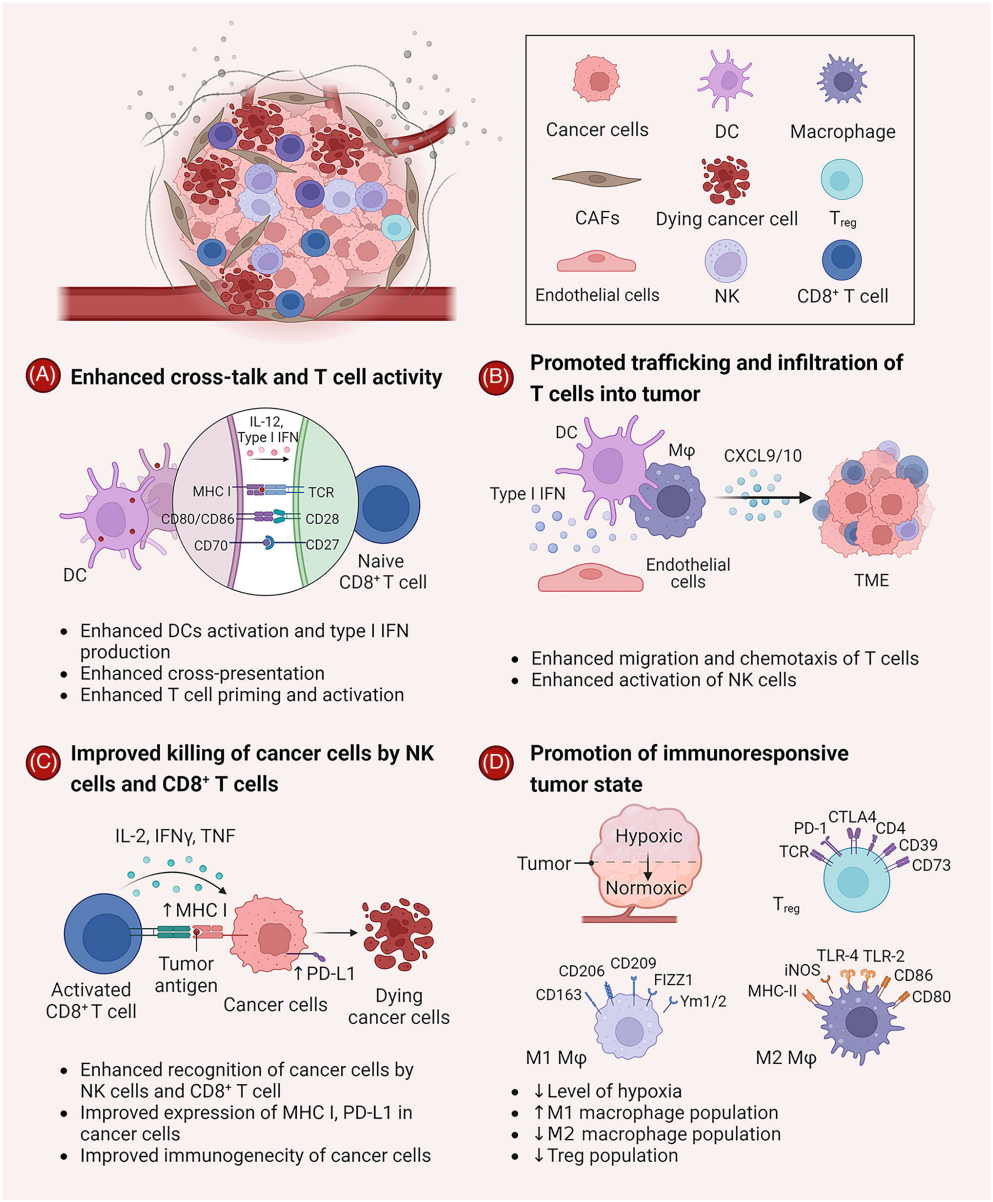
STING pathway activation can convert the tumor microenvironment from an immunosuppressive “cold” state to a proinflammatory “hot” state via the following steps: (A) improved antigen immunogenicity, processing, and presentation, and T‐cell priming, activation, thereby enhancing CD8^+^ T‐cell immunity; (B) enhanced trafficking and infiltration of T cells into tumor area by induced chemokines due to increased type I IFN; (C) enhanced killing of tumor cells by NK cells and CD8^+^ T cells; (D) converting to normalized TME including blood vessel normalization, decreased level of hypoxia within the tumor, suppression of immunosuppressive cell types and induction of acute inflammation by inducing the expression of various cytokines and chemokines. Figure was created with biorender.com.

In light of the bridging role of STING signaling between innate and adaptive immunity in response to DAMPs and PAMPs, a wealth of studies have reported the underlying mechanisms by which the cGAS–STING signaling pathway controls tumor progression. A common conclusion is that cGAS–STING signaling regulates multiple steps in antitumor immunity and ameliorates the immunosuppressive TME‐related factors (Figure 5).^247^, ^257^, ^258^

Activation of the STING pathway in tumor cells can directly induce tumor cell senescence or various death modalities (such as apoptosis, necroptosis, ferroptosis, etc.) in a cell‐autonomous manner. This cGAS–STING signaling‐pathway‐mediated tumor cell suppression is thought to be mediated through the regulation of type I IFN (IFN‐α and IFN‐β) and other proinflammatory genes.^259^ Evidence suggests that cGAS–STING signaling‐pathway‐mediated autophagic tumor cell death occurs through the formation of micronuclei upon sensing aberrant cell fusion.^260^ During the autophagy process, the STING protein acts as both an autophagy substrate and a modulator of autophagy, and a potential mechanism is via autophagosome cargo protein sequestosome 1(SQST)‐mediated signals.^261^ Notably, studies suggest that DNA damage under specific conditions triggers the release of DNA (mitochondrial DNA and nuclear DNA) into the cytosol, leading to the activation of STING signaling and subsequent autophagy‐dependent ferroptosis in both mice and human pancreatic cancer cells. These responses have also been found to result in tumor growth control in associated murine pancreatic cancer models.^262^ Moreover, the activation of STING signaling is reported to be involved in the induction of tumor cell apoptosis through the IRF3–BCL2‐associated X protein/BCL2‐antagonist/killer 1 pathway and necroptosis by triggering the expression or phosphorylation of mixed‐lineage kinase domain‐like pseudokinase, one of the major regulators of necrosome formation during necroptotic cell death.^263^, ^264^ Interestingly, in contrast to the proapoptotic effect in tumor cells, activation of the STING pathway in DCs and macrophages is antiapoptotic.^265^, ^266^

### 3.2 Role of cGAS–STING pathway on immune cells in TME

Through enhancing the immunogenicity of tumor cells by ICD and promoting the maturation of APCs, thereby initiating CD8^+^ T‐cell responses, STING activation results in improved therapeutic efficacy.^267^, ^268^, ^269^, ^270^ Recent evidence supports the notion that, except for tumor‐cell‐produced single‐stranded DNA (ssDNA) or dsDNA (e.g., induced by genomic instability), DNA damage due to chemotherapy or radiotherapy and abnormal tumor cell proliferation can also directly induce DC maturation and recruitment via STING activation.^271^, ^272^ In addition, cGAMP amplifies the capacity of neighboring DCs to produce type I IFN and related cytokines by transfer via EVs secretion or gap junctions.^273^ Research has demonstrated that the initiation of spontaneous tumor antigen‐specific T cells is reliant on DC activities and the production of type I IFN.^274^ Intratumoral injection of STING agonists has been shown to enhance CTLs infiltration into tumors.^275^, ^276^ Because STING activation and type I IFN secretion within the TME upregulated the expression of genes encoding inflammatory cytokines (IL‐12, TNF‐α, IFN‐γ, etc.) and T cell‐recruiting chemokines (C‐X‐C motif chemokine ligand [CXCL]9 and CXCL10), thereby transforming immunosuppressive “cold” tumors into T‐cell‐inflammatory “hot” tumors.^277^, ^278^, ^279^ Additionally, STING agonists can directly activate T cells, independent of DC‐released type I IFN. However, activation of the STING pathway in T cells may result in impaired T lymphocyte proliferation, reduced memory cell counts, and activation of the T cell emergency and death pathways, ultimately leading to T cell death.^280^, ^281^

STING activation can also increase the sensitivity of tumor cells to immune NK cells and CTLs.^282^ However, careful consideration is still needed to explore the impact of STING activation on NK‐cell function, as one recent publication showed that excessive STING activation in B cells might diminish NK‐cell function. In addition, STING pathway activation can also promote TME normalization by regulating macrophage polarization.^283^ Briefly, STING agonists repolarize tumor‐associated macrophages from the M2 type towards the M1 type through NF‐κβ/IRF3 activation and type I IFN upregulation.^284^, ^285^ It has been reported that systemic or intratumor administration of STING agonists reverses the immunosuppressive TME and contributes to tumor regression.^286^

### 3.3 Role of cGAS–STING pathway on endothelial cells in TME

In addition to immune cells, recent research has identified stromal cells, particularly endothelial cells, as a target of the STING pathway within the TME.^276^, ^287^, ^288^, ^289^ The initial STING agonists (DMXAA, 5,6‐dimethylxanthenone‐4‐acetic acid) have demonstrated the ability to act as vascular disruptors, thereby inhibiting tumor growth.^290^ Activation of STING within endothelial cells has been shown to promote the secretion of IFN‐β, which in turn leads to T‐cell infiltration into solid tumors. The blockade of IFN signaling using IFNAR antibody or IFNAR ablation can further eliminate T‐cell infiltration.^276^ Adhesion to endothelial cells is a crucial step in the process of T‐cell infiltration.^291^ Intratumoral injection of STING agonists into tumor‐bearing mice (cGAMP or ADU‐S100) upregulated type I/II IFN genes, vascular stabilizing genes (including *Angpt1*, *Pdgfrb*, and *Col4a*), and adhesion molecules (including *Icam*, *Vcam*, and *Sell*), promoted tumor vascular normalization, reduced tumor hypoxia, and increased tumor‐specific CTL infiltration.^287^ However, in VEGF‐rich cancers, VEGF/VEGFR2 signaling might hamper the antitumor efficiency of STING agonists due to its negative regulation of type I IFN signaling activation via ubiquitin‐mediated IFN α and β receptor (IFNAR) degradation.^292^, ^293^ Additionally, VEGF (and other cytokines) secreted by cancer cells may suppress the expression of molecules that mediate T cell adhesion on endothelial cells or induce the expression of molecules that trigger the death of effector T cells.^294^ Related to this, administration of STING agonists together with antiangiogenic/antivascular agents as well as an ICI showed the potential to promote T‐cell infiltration into the TME and prevented tumor recurrence in a colorectal mouse model.^287^ Careful consideration needs to be given to the combination of STING agonists with VEGF blockers, as this may inhibit T‐cell infiltration by suppressing the proliferation of endothelial cells within the tumor, leading to the progression of certain cancer types. This latter is because appropriate levels of VEGF are necessary to maintain endothelial cell populations.^295^ It can be hypothesized that targeted lipid‐NPs might address this issue through increasing specificity to reduce the uptake of VEGF blockade agents by endothelial cells. However, the effectiveness and possibility of targeted lipid‐NPs for STING and VEGF co‐therapy in VEGF‐rich cancer types still needs to be explored.

## 4 TARGETING THE cGAS–STING SIGNALING PATHWAY

### 4.1 Targeting the cGAS–STING signaling pathway in cancer clinical trials

**FIGURE 6:**
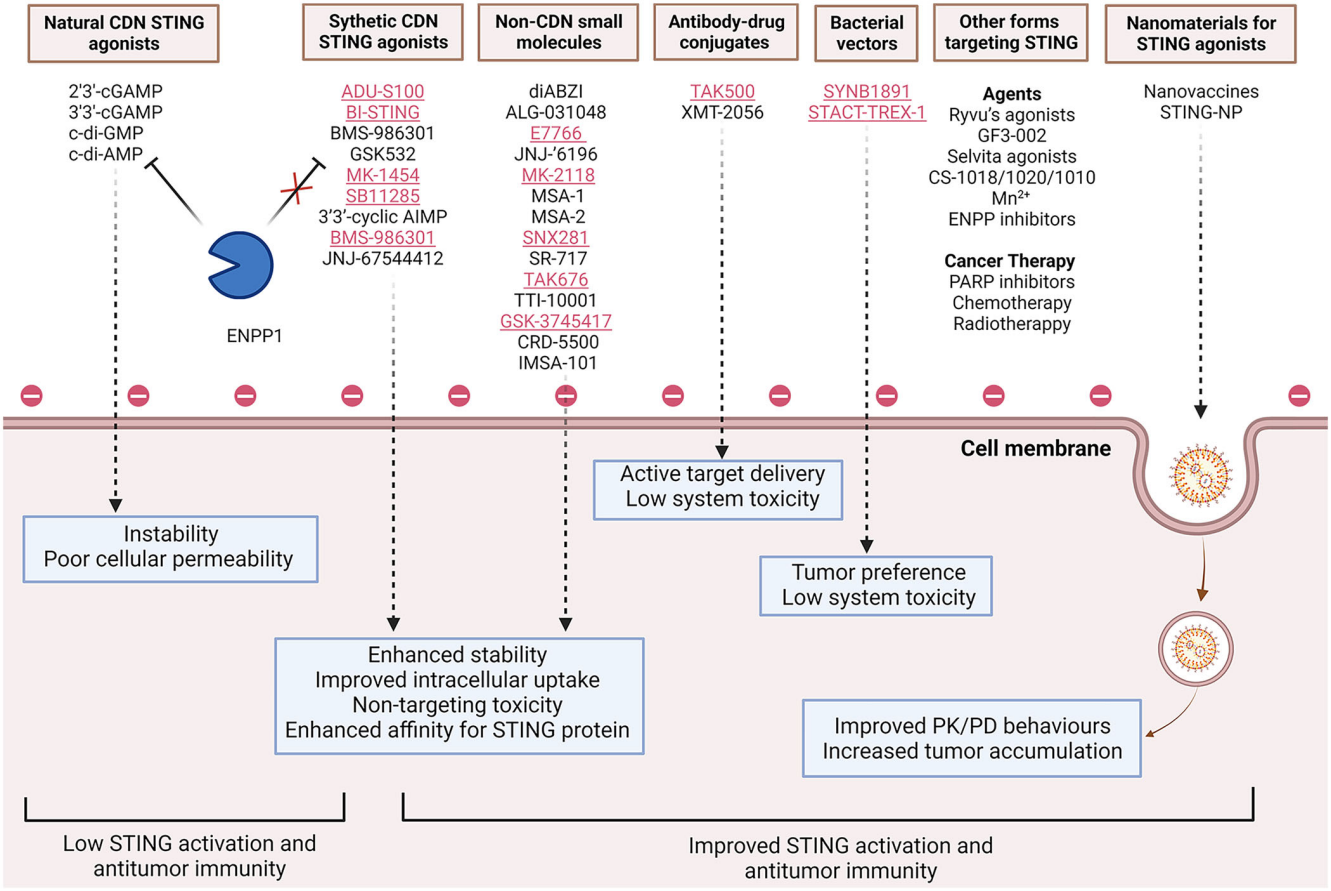
Targeting the cGAS–STING signaling pathway in cancer treatment. Various STING agonists and other forms of therapies targeting the cGAS–STING pathway are depicted. Agonists undergoing clinical trials are indicated with red font, and agonists undergoing preclinical development are indicated with black font. The minus signs in circles above the cell membrane represent negative charge. Figure was created with biorender.com.

The STING signaling pathway has emerged as a therapeutic target in tumor therapy (Table 3). As the first STING agonist, DMXAA was created and investigated for its anticancer therapeutic benefits. Multiple preclinical studies have indicated that intratumoral or systemic delivery of DMXAA might stimulate the innate immune system, inhibit tumor angiogenesis, and exhibit tumor suppression in various types of cancer (e.g., NSCLC, breast cancer, melanoma, fibrosarcoma, etc.).^285^, ^290^, ^296^, ^297^, ^298^ However, the clinical outcomes of DMXAA treatment have not been satisfactory, due to the strong affinity of DMXAA for mouse STING protein but not for human STING.^296^, ^299^, ^300^ Inspired by the potent antitumor immunity induction of DMXAA, various agonists targeting human STING signaling, including eukaryotic/prokaryotic STING ligands and synthetic mimics, have been developed: CDNs, non‐CDN small molecules, nanovaccines, antibody–drug conjugates (ADCs), and so on (Figure 6).^301^ However, the stability and efficiency of natural STING agonists, such as 2′3′‐cGAMP, 3′3′‐cGAMP, cyclic di‐AMP (CDA), and cyclic di‐GMP (cdGMP), render STING‐targeted therapy a challenge.^302^ Additionally, synthetic CDNs have been developed to circumvent the limited half‐life and stability of natural CDNs, and they have shown variable results. For example, ADU‐S100 (ML‐RR‐S2, MIW815) is a synthetic CDN that explicitly targets human STING signaling with enhanced stability and affinity, and has been used in the treatment of advanced and metastatic solid cancer.^285^ In clinical settings, the development of ADU‐S100 has been put on hold due to the limited antitumor immunity conferred by single treatment or in combination with various ICIs, including anti‐programmed death‐1 (PD‐1) antibodies and anti‐cluster of differentiation 152 (CTLA‐4) antibodies (clinical trials: NCT02675439, NCT03172936, NCT03937141). A completed Phase 1 study (clinical trial: NCT03010176) showed that 6 of 25 patients responded to the combination therapy of MK‐1454 (a synthetic CDN) and pembrolizumab (anti‐PD‐1 antibodies), whereas no significant response was observed as a result of MK‐1454 monotherapy.^303^ Additionally, a self‐assembled CDN, SB11285, has been shown to improve physicochemical properties against enzymatic degradation, and its clinical development as a monotherapy or in combination with atezolizumab is in progress (anti‐PD‐L1 antibodies, clinical trials: NCT04096638).

**TABLE 3:** Clinical trials of STING agonists in cancer.

Phase	Agonist	Cointervention	Cancer type	Status	Clinical trial reference number
Phase I/II	CDK‐002/exoSTING (i.t.)	–	Advanced/metastatic, recurrent, injectable solid tumors	Completed (plan to into Phase 2 in bladder cancer)	NCT04592484
	IMSA101 (i.t.)	Immune checkpoint inhibitor (ICI) or Immuno‐oncology (IO) therapy	Advanced treatment‐refractory malignancies	Recruiting	NCT04020185
Early Phase I	TAK‐676 (CIVO^®^ i.t.)	MK‐0482 + pembrolizumab or MK‐4830 + pembrolizumab	Solid tumors	Recruiting	NCT04541108
Phase I	SNX281 (i.v.)	Pembrolizumab (i.v.)	Advanced solid tumors and lymphoma	Withdrawn (no enrolment of participants)	NCT04609579
	E7766 (i.v.)	–	Nonmuscle invasive bladder cancer	Withdrawn (no enrolment of participants)	NCT04109092
	E7766 (i.t.)	–	Advanced solid tumors Lymphomas	Completed (no results posted)	NCT04144140
	MK‐1454 (i.t.)	Pembrolizumab (i.v.)	Solid tumors Lymphoma	Completed (results submitted)^310^	NCT03010176
	MK‐2118 (i.t./s.c.)	Pembrolizumab (i.v.)	Advanced/metastatic solid tumors or lymphomas	Completed (no results posted)	NCT03249792
	ADU‐S100 (i.t.)	PDR001 (i.v.)	Advanced/metastatic solid tumors Lymphomas	Terminated (sponsor's decision)	NCT03172936
	ADU‐S100 (i.t.)	Ipilimumab (i.v.)	Advanced/metastatic solid tumors Lymphomas	Terminated (no substantial antitumor activity was observed.)	NCT02675439
	KL340399 (i.t.)	–	Advanced solid tumors	Recruiting	NCT05549804
	KL340399 (i.v.)	–	Advanced solid tumors	Not yet recruiting	NCT05387928
	TAK‐500 (i.v.)	Pembrolizumab (i.v.)	Locally advanced Metastatic solid tumors	Recruiting	NCT05070247
	TAK‐676 (i.v.)	Pembrolizumab (i.v.) or Pembrolizumab (i.v.) Carboplatin/cisplatin (i.v.) 5‐Fluorouracil (i.v.)	Advanced or metastatic solid tumors	Recruiting	NCT04420884
	TAK‐676 (i.v.)	Pembrolizumab (i.v.) Radiation therapy	Non‐small‐cell lung cancer Triple negative breast cancer Head and neck squamous cell carcinoma	Recruiting	NCT04879849
	GSK3745417 (administration route was not specified)	–	Leukemia	Recruiting	NCT05424380
		Dostarlimab (i.v.)	Neoplasms	Active, not recruiting	NCT03843359
	BMS‐986301 (i.m./i.v./i.t.)	Nivolumab (i.v.) Ipilimumab (i.v.)	Advanced solid cancers	Active, not recruiting	NCT03956680
	SB11285 (i.v.)	Atezolizumab (i.v.)	Melanoma Head and neck squamous cell carcinoma Solid tumor	Recruiting	NCT04096638
	BI 1387446 (i.t.)	Ezabenlimab (i.v.)	Advanced or metastatic cancer	Active, not recruiting	NCT04147234
	SNX281 (i.v.)	Pembrolizumab (i.v.)	Advanced solid tumors Advanced lymphoma	Recruiting	NCT04609579
	SYNB1891 (i.t.)	Atezolizumab (i.v.)	Advanced/metastatic solid tumors Lymphom	Recruiting	NCT04167137
	ONO‐7914 (administration route was not specified)	Nivolumab (i.v.)	Solid tumors	Recruiting	JRCT2031210530
	HG381 (administration route was not specified)	–	Advanced solid tumors	Recruiting	NCT04998422
	DN1508052‐01 (s.c.)	–	Neoplasms	Completed	NCT03843359
	KL340399 (administration route was not specified)	–	Advanced solid tumors	Not yet recruiting	NCT05387928
	STING‐dependent Activators (STAVs, i.v.)	Dendritic cell vaccine	Aggressive relapsed/refractory leukemias	Not yet recruiting	NCT05321940
Phase II	ADU‐S100 (i.t.)	Pembrolizumab (i.v.)	Head and neck cancer	Terminated (no substantial antitumor activity was observed.)	NCT03937141
	MK‐1454 (i.t.)	Pembrolizumab (i.v.)	Head and neck squamous cell carcinoma	Completed (no results posted)	NCT04220866

i.t., intratumorally; i.v., intravenously; s.c., subcutaneously; ND, not described. (Data sources —clinical registration website: www.clinicaltrials.gov)

Non‐CDN small molecules for STING pathway activation are currently under clinical investigation. E7766, a topologically novel macrocycle‐bridged STING agonist, has been used to treat nonmuscle invasive bladder cancer (NMIBC).^304^ Due to insufficient enrolment of participants, this Phase I study was unfortunately terminated in 2020 (clinical trial: NCT04109092). However, another clinical trial, NCT04144140, has since started, aiming to determine the safety profile and efficacy of E7766 as a single agent for intratumoral administration in patients with advanced solid tumors or lymphomas. Both TAK‐676 and MK‐2118 are small‐molecular STING agonists. Currently, both molecules are undergoing clinical assessment as monotherapies or in combination with pembrolizumab for evaluation of their safety and anticancer effects for advanced/metastatic solid tumors (clinical trials: NCT04420884, NCT03249792). HG381 is a non‐CDN small molecule STING agonist developed on the basis of on DNA‐encoded compound library (DEL) technology and small molecule new drug development platform, currently in clinical Phase I (clinical trial: NCT04998422) but no relevant clinical and preclinical data have been released. Another non‐CDN small molecule compound is KL340399, that is presently in Phase I clinical study for the treatment of advanced solid tumor indications through intravenous injection and for superficial or locally advanced, recurrent or metastatic solid tumors. This compound can be administered with the aid of medical imaging instruments via intratumoral injection (clinical trial: NCT05387928).

Multiple ADCs have been developed as therapeutic modalities for cancer with enhanced selectivity to tumor cells and reduced toxicity to normal cells.^305^ Inspired by the promising results of the synthetic STING agonist TAK‐676 in Phase 1 clinical trials (NCT04420884, NCT04879849), researchers have designed a novel STING agonist termed TAK500 by linking TAK‐647 to a cysteine–cysteine chemokine receptor type 2 (CCR2) antibody via a protease‐cleavable peptide.^306^, ^307^ When incorporated into tumor‐infiltrating CCR2^+^ myeloid cells, this may lead to the activation of STING/IFN signaling and remodel the TME, thereby eliciting antitumor immunity. Currently, TAK‐500 is being evaluated in a Phase I trial for safety, tolerability, and anticancer efficiency, and investigation of its combination with the anti‐PD‐1 blockade antibody for the treatment of late‐stage cancer patients is ongoing (clinical trial: NCT05070247). Another example is the combination of XMT‐2056 (a small‐molecular molecule) and trastuzumab (a mAb targeting human epidermal growth factor receptor [HER] 2 receptor) for boosting host immunity against HER2‐expressing solid tumors; XMT‐2056 has been granted orphan drug status for patients with gastric cancer. A clinical trial of XMT‐2056 is in progress.^308^, ^309^

### 4.2 Targeting the cGAS–STING signaling pathway in cancer preclinical studies

In preclinical studies targeting the cGAS–STING signaling pathway, many CDN surrogates have also been developed to enhance antitumor immune responses. For example, various specific metal ions such as manganese ion (Mn^2+^) and lanthanides have been observed to activate the STING pathway and that Mn^2+^ self‐assembled into NPs (CDN‐Mn^2+^ particles, CMP) in concert with CDN STING agonists can initiate potent antitumor immunity at microdoses.^311^, ^312^ The same STING activation ability has been observed with europium‐based nanovaccines that are composed of GMP, AMP, and coordinating lanthanides.^313^ In addition, more non‐CDN small‐molecule STING agonists are being developed. For example, through high‐throughput in vitro screening, a small molecule named G10 (4‐(2‐chloro‐6‐fluorobenzyl)‐N‐(furan‐2‐ylmethyl)‐3‐oxo‐3,4‐dihydro‐2H‐benzo[b][1,4]thiazine‐6‐carboxamide) were screened and categorized as hSTING‐specific agonists that can activate the IFN response via IRF3 in a dependent manner.^314^, ^315^ Likewise, by using engineered human liver cells HepAD38 in a bioassay that permits the large‐scale screening of compound libraries for activation of cGAS–STING pathway, a bis‐spirocyclic diketopiperazine compound that induces cytokine responses in a manner dependent on functional human STING expression.^316^ It has been reported that α‐mangostin, which bears the xanthone skeleton, could also serve as a human STING (hSTING) agonist with the ability to remodel human monocyte‐derived M2 macrophages toward the M1 phenotype.^317^, ^318^ Similar hSTING specificity was recently observed with bicyclic benzamides and benzothiophene derivatives bearing a benzamide framework and abilities to e ability to inhibit tumor growth by anti‐immunity induced by STING‐activation.^319^ Another novel STING agonist can be linked to two amino‐benzimidazole (ABZI) compound (diABZI), which are two symmetry‐related links two symmetry‐based, has the ability to bind with STING protein and exhibits potent and lasted antitumor immunity when injected into syngeneic colon cancer mouse models.^320^, ^321^

Among those being investigated extensively for preclinical characterization are summarized in Table 4, including bacterial vectors (such as SYNB1891 and STACT‐TREX‐1), ectonucleotide pyrophosphatase/phosphodiesterase (ENPP1) inhibitors, s‐acylthioalkyl ester (SATE)‐based STING‐activation prodrugs selenium‐containing chemicals based on the structure of benzothiophene oxobutanoic acid (MSA‐2), newly developed 7‐(het)aryl 7‐deazapurine CDNs, small‐molecule STING agonist include SR‐001, SR‐012, SR‐717, SR‐301 and SR‐717, as well as novel compound named HB3089, and so on.^322^, ^323^, ^324^, ^325^, ^326^ These preclinical small molecular STING agonists have been recently extensively reviewed by Garland et al.,^327^ and will therefore not be discussed in detail in this review.

**TABLE 4:** Preclinical studies on STING‐targeting compounds.

Agents	Types	Target	Route of delivery	Cancer models	Cytokines or chemokines	Immune cells	References
GSK532	CDN	hSTING and STING orthologs from cyno, minipig, dog, rat, and mice	i.t.	CT26 murine tumor model	TNF‐α IL‐6	ND	^328^
JNJ‐67544412 (JNJ‐4412)	CDN	hSTING mSTING	i.t.	Subcutaneous syngeneic murine tumor models	IFN‐α IFN‐β IP‐10 TNF‐α IL‐6 MCP‐1	CD8^+^ T cells	^329^
3′3′‐cyclic 3′3′‐cAIMP	CDN	hSTING	ND	Mutagen diethylnitrosamine (DEN) induced murine hepatocellular carcinoma	IFN‐α TNF‐α CCL5 CXCL9 CXCL10	CD3+ T cells CD8+ T cells	^330^
7‐Deaza Variants	CDN	hSTING	ND	ND	ND	ND	^324^
Ryvu's agonists	Non‐CDN small molecule compounds	hSTING mSTING	i.v.	CT26 and EMT6 murine tumor model	ND	ND	^331^, ^332^
Selvita agonists	Non‐CDN small molecule compounds	hSTING mSTING	Not applicable	In vitro	IFN‐β TNF‐α	DCs Macrophages	^333^
CRD5500	Non‐CDN small molecule compounds	hSTING	i.t.	CT26 murine tumor model	IFN‐β TNF‐α	DCs	^334^
			i.v.	B16F10, PANO2, LL2 and C1498 murine tumor models	IFN‐β TNF‐α CXCL10	DCs	^335^
GF3‐002	Non‐CDN small molecule compounds	hSTING	Not applicable	In vitro	IFN‐β	DCs	^336^
JNJ‐‘6196	Non‐CDN small molecule compounds	hSTING	i.v.	Murine tumor models	ND	DCs Macrophages CD8+ T cells	^337^
TTI‐10001	Non‐CDN small molecule compounds	hSTING mSTING	i.t.	Multiple syngeneic murine tumor models	IFN‐β TNF‐α IL‐6	ND	^338^
CS‐1018 CS‐1020 CS‐1010	Non‐CDN small molecule compounds	hSTING mSTING	i.t.	B16F10 and MC38 murine tumor models	ND	ND	^339^
MSA‐1	Non‐CDN small molecule compounds	hSTING mSTING	i.t.	B16F10, MC38, and CT26 mouse tumor models	ND	T cells memory T cells	^340^
MSA‐2	Non‐CDN small molecule compounds	hSTING	Oral administration	B16F10, MC38, LL2, and CT26 murine tumor models	ND	ND	^326^
VB‐85247	Macrocyclic STING agonists	hSTING mSTING	Bladder instillation	MB49 and NMIBC mouse tumor model	type I IFN	DCs	^341^
ALG‐031048	Novel STING agonist, Similar to 2'‐3'‐cGAMP	hSTING mSTING	i.t. s.c.	CT26, B16F10, and Hepa1‐6 mouse tumor models	IFN‐β CXCL10	Macrophages	^342^
SR‐8541A	ENPP1 inhibitor	ENPP1	Not applicable	In vitro 3D spheroid models and human breast cancer cell line derived organoids; CT‐26 and EMT‐6 mouse models	IFN‐β CXCL10	NK cells DCs CD3+ T cells CD8+ T cells	^343^, ^344^
SR‐8314	ENPP1 inhibitor	ENPP1	i.p.	Syngeneic murine tumor model	IFN‐β CXCL10	CD3+ T cells CD4+/CD8+ T cells	^345^
MV‐626	ENPP1 inhibitor	ENPP1	i.p.	Panc02‐SIY and MC38 murine tumor models	ND	ND	^346^
AVA‐NP‐695	ENPP1 inhibitor	ENPP1	ND	4T1 syngeneic tumor model	ND	ND	^347^
ZX‐8177	ENPP1 inhibitor	ENPP1	i.p.	CT‐26, Panc02 and MC38 murine tumor models	IFN‐β IFN‐α CCL5 CXCL10	Granzyme B+CD8+ T cells NK cells macrophages	^348^, ^349^
LCB33	ENPP1 inhibitor	ENPP1	Oral administration	CT26 murine tumor model	type I IFN	T cells Immune cells (not specified)	^350^
ZXP‐8202	ENPP1 inhibitor	ENPP1	ND	CT26 murine tumor model	IFN‐β	ND	^351^
SB02024	VPS34 inhibitor	VPS34	Oral administration	B16F10 mouse tumor model	ND	ND	^352^
JAB‐X1800	CD73‐STING iADC	hSTING mSTING	ND	MDA‐MB‐231 xenograft mouse tumor model and hCD73‐MC38 syngeneic mouse tumor model	IFN‐β	ND	^353^
FcγR specific STING‐agonist ADCs	FcγR‐STING ADC	ND	Not applicable	In vitro	ND	FcγRI^+^ myeloid cells	^354^
pHLIP‐STINGa	pHLIPs (pH‐Low Insertion Peptides) (Var3) linked to a STINGa (diABZI) via a self‐immolating linker	hSTING	i.p. i.v.	CT26, and B16F10 mouse tumor models	ND	Macrophages Cancer‐associated fibroblasts (CAFs) Myeloid‐derived suppressor cells DCs T cells NK cells Memory T cells	^355^
ST317	Peptide	hSTING mSTING	i.v.	Syngeneic mouse models	type I IFN CXCL10	ND	^356^
rBCG‐ disA ‐OE	Recombinant Bacillus‐Calmette Guerin (BCG) overexpressing CDN	hSTING mSTING	ND	Nonmuscle invasive bladder cancer mouse model	TNF‐α IL‐6 IL‐1β Nos2 CCL2	ND	^357^
STACT‐TREX1	An inhibitory microRNA to TREX1 was designed and introduced into the STACT strain.	TREX1	i.v.	CT26, MC38, and B16‐F10 mouse tumor model	TNFα IL‐6	ND	^358^
STINGVAX	CDN with GM‐CSF‐producing cellular cancer vaccine	hSTING mSTING	i.v.	CT26, Panc02, and B16‐F10 mouse tumor model	IFN ‐α IL‐12 IFN‐γ IL‐6 CCL2 IFN‐β	CD4+/CD8+ T cells T helper 1 (TH1) cells	^330^
ONM‐501	pH sensitive polyvalent PC7A micelles loaded with cGAMP	hSTING mSTING	i.t. s.c.	Mice, rats, and primates	ND	ND	^359^, ^360^

i.t., intratumorally; i.v., intravenously; s.c., subcutaneously; i.p., intraperitoneally; ND, not described.

## 5 LIPID‐BASED NANOMATERIALS FOR DELIVERY OF STING AGONISTS

As discussed above, although multiple STING agonists have shown effective tumor inhibitory efficiency when given intravenously or intratumorally, their clinical translation has not been materialized. The low cytoplasmic delivery rate is due to the hydrophilic and negative electrical properties of exogenous DNA and CDNs, while the rapid immune clearance and noncellular targeting of natural and synthetic CDNs limit the intracellular activation of the STING pathway.^302^ Moreover, relatively high concentrations of STING agonists are needed to elicit sufficient STING signaling activation when administrated intravenously, and probably lead to systemic inflammatory toxicity.^18^ To achieve optimal clinical feasibility, the response efficiency of STING agonists still needs to be improved. There is growing evidence indicating that appropriate combination therapy regimens based on STING agonists could expand the therapeutic benefits of STING agonists for cancer patients. Another promising method for improving the efficiency of STING agonists is the use of NPs for the delivery of STING agonists, following the rationale of improving the efficacy and safety of intracellular delivery.^18^

### 5.1 Liposomes for STING agonist delivery

Liposomes, especially PEGylated liposomes, as delivery vehicles for STING agonists or other agents capable of activating cGAS–STING pathway can increase their stability, prolong the blood circulation time, enhance tumor targeting, and promote intracellularization, thus maximizing the antitumor immune effect and reducing adverse effects.^19^ As described in a study by Koshy et al.,^361^ 24 h after delivery of non‐PEGylated and PEGylated cGAMP liposomes, cGAMP delivered by PEGylated liposomes aggregated in the whole tumor site rather than within small tumor volumes, as was the case for non‐PEGylated liposome delivery; treatment with PEGylated cGAMP liposomes resulted in a 50% tumor ablation and a complete rejection upon tumor rechallenge in the melanoma mouse model, which was shown unresponsive to non‐PEGylated cationic liposome challenge. In addition, by modifying the percentage of PEG surface modification, liposomes can be fine‐tuned to optimize their serum stability and surface charge properties; the cGAMP liposome formulation with 5 mol% PEG induced a better adaptive immune memory response than the formulation with 10 mol% PEG.^361^ The rational structural fine‐tuning through modifications to the liposome surface, such as specific antibodies, peptides, or receptor ligands, can also enhance the biological effects of STING agonists.^362^, ^363^, ^364^, ^365^ For instance, mannose‐modified liposomes encapsulating STING ligands (2′‐3′‐cGAMP) with a diameter of 70 nm and a surface charge of about 7 mV were able to specifically target APCs (both DCs and macrophages) and showed increased stimulation of the STING pathway. This approach significantly promoted the transition of the immunosuppressive TME towards an immunostimulatory state as compared with treatment with free cGAMP in the V600E BRAF‐mutated (an activating missense mutation in codon 600 of exon 15 (V600E) of the B‐Raf proto‐oncogene [BRAF] gene) melanoma mouse model.^366^Activation of DCs is a prerequisite for induction of antitumor immunity. Doshi et al.^367^ reported a CD103^+^ DC‐targeted STING‐liposomes, which selectively deliver STING agonists (CDN) into DCs, induce robust antitumor immune responses at a low dose, resulting in improved immunotherapy efficiency and reduced side effects in MC38 and B16F10 tumor models. Additionally, Kocabas et al.^368^ examined the immunostimulatory capacity and antitumor potential of pH‐sensitive cationic liposomes codelivered with a STING agonist and other immune adjuvants in a mouse model of melanoma. The liposomes, prepared using DOPE, DC‐chol, PEG‐PE, PC, cholesterol, and cGAMP, had a size of approximately 400–700 nm with stable characteristics under neutral conditions and rapidly destabilized properties in acidic environments (lysosome or late endosome). The cGAMP‐loaded formulation, when codelivered with CpG ODN, induced splenocytes to produce more IFN‐α/β than free cGAMP, and significantly inhibited B16 melanoma growth through re‐education of the TME towards a tumor‐suppressive state and promotion of Th1 immune response.^368^ In another study, tumor‐penetrating neutrophils cytopharmaceuticals with STING agonists were developed. In brief, they prepared a cationic liposomal STING agonists (DMXAA) coated with negative HA‐maleimide (HA‐Mal) and conjugated these onto the surface of neutrophils, named NEs@STING‐Mal‐NP.^369^ The NEs@STING‐Mal‐NP has the ability of penetrating the tumor vascular endothelium and quick STING agonist release. These characteristics endowed NEs@STING‐Mal‐NP with an increased absorption rate by immune cells and tumor cells, resulting in an antitumor TME stated via macrophages and neutrophils phenotype education and T cell infiltration improvement.^369^

### 5.2 CLD NPs for STING agonist delivery

Cationic LNPs appear more promising for realizing the full potential of STING agonists as they may induce stronger immune responses than anionic or neutral liposomes, due to alkalinizing lysosomal pH and limitation of antigen degradation.^370^, ^371^, ^372^ For example, STING agonists delivered by a system composition of a neutral cytidinyl lipid (DNCA) and a CLD enable robust antitumor effectiveness at a lower dosage, because it can easily enter cells and release STING agonists into the cytoplasm.^19^ Moreover, Koshy et al.^361^ showed that cholesterol‐stabilized LNPs based on DOTAP, with a size of approximately 160 nm, significantly enhanced the cell association and internalization of 2′3′‐cGAMP in bone‐marrow‐derived dendritic cells (BMDCs) compared with free cGAMP, and enhanced the biological effects of cGAMP on activation of the STING pathway. Unfortunately, intratumoral injection of low doses of cGAMP‐loaded LNPs (0.35 μg × 2 doses) did not show superior antitumor effects in an orthotopic B16‐F10 melanoma model due to limited tumor penetration. Therefore, additional efforts are required to increase the stability and tumor penetration of this formulation.^361^ A typical strategy was provided by Nakamura et al.,^373^ who devised a PEGylated STING agonist LNPs based on YSK12‐C4 (an ionizable CLD with high affinity for immune cells), PEG2000‐DMG, cholesterol, and c‐di‐GMP, which is 170 nm in size and has a 7 mV zeta‐potential. This PEGylated cationic LNPs remained stable at 4°C for up to 9 months, maintaining its ability to stimulate IFN‐β production. Upon intravenous injection, the STING‐LNPs treatment induced a superior antitumor effect in a B16‐F10‐luc2 lung metastatic mouse model.^373^ Furthermore, Miyabe et al.^374^ tried to effectively control the release of the STING agonist c‐di‐GMP at the tumor site in response to changes in pH; they used a synthetic pH‐sensitive YSK05 lipid with high fusogenic properties, together with palmitoyl‐oleoyl‐phosphoethanolamine, 1,2‐dimyristoyl‐sn‐glycerol (DMG)‐PEG2000, and cholesterol to create a liposomal formulation with a size of approximately 170–180 nm and a potential of around −5 mV. This vehicle showed high fusion and endosomal escape ability, thus leading to a superior activation effect when inducing APC activation and maturation, NK‐cell activation, and T‐cell immune response when compared with free delivery or conventional cationic liposome delivery of c‐di‐GMP. Importantly, c‐di‐GMP/YSK05 liposomes carried with tumor antigens (OVA) exhibited significantly enhanced vaccination effects in a lymphoma mouse model compared with free c‐di‐GMP+OVA, and decreased lung metastasis of melanoma in a B16‐OVA mouse model.^374^, ^375^

With high‐throughput screening using a combinatorial library of liposomal units (cations, linker molecules, and alkyl chains), Miao et al.^376^ recently found that cationic LNPs with a cyclic amine moiety, a dihydroimidazole linker molecule, and a structure of unsaturated lipid chains were able to activate the STING pathway, induce systemic cytokine expression, and enhance antitumor efficacy, indicating the potential of LNPs as an effective STING agonist delivery platform. Additionally, the results showed that when LNPs were further loaded with tumor‐associated OVA mRNA, they could be used to effectively deliver mRNA into lymphoid tissues and stimulate the STING pathway, thus producing a potent antitumor immune response and superior tumor growth inhibition in B16F10‐OVA melanoma mice.^377^ This work provides a theoretical basis for the codelivery of multiple antigens and STING agonists by LNPs. More follow‐up studies need to be conducted to clarify the full potential of nanoscale mRNA vaccines based on STING agonists. On the other hand, the selectivity and efficiency of the delivery of STING‐LNPs to different types of immune cells and different TME stages can be adjusted by modifying the targeting ligands on the surface of the LNPs. Covarrubias et al.^377^ compared the effects of common targeting ligands (the c(RGDfC) peptide for the targeting of αvβ3 integrins, the CDAEWVDVS peptide for the targeting of P‐selectins, and the CREKA peptide for fibrin targeting) on the selectivity and antitumor efficiency of the 60 nm STING‐LNPs containing various PLs: DPPC, DOPC, DSPE–PEG–ligand, and cdGMP. In a 4T1 mouse tumor model, nontargeted STING‐LNPs were found to be more aggregated in primary tumors and sites of late metastasis, while targeted STING‐LNP variants, especially integrin‐targeting cRGD‐LNPs, accumulated more in early metastases. It might also be interesting to employ more specialized targeting ligands (e.g., CCR2, mannose and intercellular adhesion molecule‐1) for LNP‐based cytoplasmic delivery of STING into APCs.^377^

### 5.3 Cellular‐membrane‐derived vesicles for STING agonist delivery

As discussed earlier, EVs, including exosomes, microvesicles, and so on, have been used and engineered as NVs to carry drugs for cancer therapies because of their ideal safety and stability properties.^378^, ^379^, ^380^ The use of such NVs for STING agonist delivery is an attractive approach, because the unique abilities of cellular vesicles inherited from particular source cell types are able to enhance the selectivity and antitumor efficiency of STING agonists.^146^ For example, Rao et al.^381^ extracted platelet‐derived NVs, M1 macrophage‐derived NVs, and cancer‐cell‐derived NVs overexpressing high‐affinity signal regulatory protein (SIRP)α variants (SαV‐C‐NVs) and formed hybrid cellular membrane NVs, with a size of 100 nm and encapsulating CDNs (hNVs@cGAMP), through repeated extrusion and ultrasound treatments. The hNVs@cGAMP blocked the antitumor inhibition of the CD47 signaling pathway and promoted the repolarization of tumor‐associated macrophages. Upon intravenous administration, hNVs@cGAMP effectively improved the cytosolic delivery of the drug owing to its specific membrane anchoring and transmembrane‐protein‐mediated endocytosis, and exhibited better inhibition of 4T1 breast tumor growth, recurrence, and lung metastasis compared with hNVs + cGAMP.^381^ In addition, exosomes derived from HEK293 cells loaded with a small‐molecule STING agonist (named exoSTING), which is designed to allow delivery directly to APCs, were shown a hundredfold more potent than free STING agonists by delivering drugs into the cytosol, overcoming the bell‐shaped pharmacological properties of STING adjuvants and increasing their tumor retention.^382^ Of note, exoSTING is the only liposomal STING agonist so far that is being tested in clinical studies (NCT04592484). Related data has been reported in June 2022 and the company Codiak Biosciences plans to take exoSTING into a Phase 2 study for bladder cancer patients next year (Source: Codiak BioSciences, Inc.; https://ir.codiakbio.com/node/7416/pdf). Similarly, engineered EVs derived from HEK293 cells were used to encapsulate STING agonists ranging in size from about 50 to about 200 nm, and this was found to reduce systemic inflammation and enhance the biological ability of the STING agonists. These data showed that EVs containing STING agonists increased DC and monocyte activation in vitro and improved tumor growth suppression in B16F10, CT26, and EG7‐OVA mouse models with a more than 100‐fold increase in potency compared with free CDNs.^383^ Furthermore, a dual‐targeting cGAMP@Exos exhibit antitumor immunity and suppressed immune escape through twice activation of DCs (cGAMP and anti‐CD40) as well as the blocking of PD‐L1 on tumor cells.^174^

### 5.4 Other lipid‐based formulations for STING agonist delivery

Lipid nanodiscs (LNDs) consist of disc‐like lipid bilayers stabilized by amphiphilic biomolecules (termed membrane scaffold proteins) and have attracted extensive attention for the delivery of drugs.^384^, ^385^, ^386^ Recently, Dane et al.^387^ conjugated CDN prodrugs with PEG‐PL via thiol‐maleimide coupling (CDN–PEG–lipid), which, upon being internalized by cells, released active STING agonists upon peptidase cleavage. Subsequently, CDN‐functionalized LNDs were self‐assembled with CDN–PEG–lipids, PEGylated lipids (PEG5000lipid), and high Tm (melting temperature) PLs. Compared with the conventional liposome–CDN structure with a diameter of approximately 60 nm, the prepared LND–CDN had more uniform and nanoscale sizes (20–30 nm) and unique structural shapes, leading to improved cellular internalization and superior tumor penetration both in vitro and in vivo. The latter was because LND–CDN easily migrated towards the vessel walls in the blood circulation and presented an increased number of binding areas to the endothelium.^388^ After intravenous injection, LND–CDN showed a significantly extended plasma half‐life of 12.6 h as compared with the rapid clearance (half‐life around 1 h) of the free soluble drug and the moderate terminal half‐life of liposome–CDN (7.6 h).^388^ Furthermore, LND–CDN demonstrated enhanced CDN delivery to tumor cells and was able to boost T‐cell immune responses through DC priming when compared with both no treatment and the liposome–CDN treatment. At a dose 20 times lower than that of the free STING ligand, a 75% tumor ablation rate was observed in the MC‐38 colorectal mouse model after LND–CDN therapy, while it was almost three times higher than that of the liposome–CDN group.^388^ Although LND may be a promising platform for the delivery of STING agonists throughout solid tumors, its translation to clinical application still requires further effort and consideration, such as with respect to optimizing LND storage and reducing the cost of synthesis.

Lipidoids, a class of lipid‐like materials, have also been investigated for the delivery of STING agonists. Chen et al.^389^ demonstrated that a 93‐O17S/cGAMP lipidoid consisting of 93‐O17S, cholesterol, DOPE, and STING ligand was able to facilitate cGAMP release within the cytoplasm of APCs by means of the endo‐/lysosomal escape effect and enhanced activation of the STING pathway in the APCs. The lipidoid 93‐O17S was chosen because of its cationic properties, which are favorable for STING agonist encapsulation, and high adjuvant ability after immunization in mice.^390^ Another high‐density lipoprotein (LV‐sHDL), loaded with a STING agonist (vadimezan) and a multitargeted tyrosine kinase inhibitor (TKI; Lenvatinib), and consisting of DMPC and cholesterol oleate, appeared as spherical NPs with a diameter of 15 nm and a negatively charged ζ‐potential (about −20 mV).^391^ This synthetic dual‐drug lipoproteins LV‐sHDL was able to enhance the tumor accumulation of the encapsulated drugs and the transport of the drugs into the cytosol of the cells via scavenger receptor class B type 1 (SR‐B1) receptors, which are consistently highly expressed on breast cancer cells.^392^ Moreover, in vivo data have shown the promising effects of LV‐sHDL on primary tumor growth control and the reduction of lung metastasis in an aggressive 4T1 triple‐negative breast cancer mouse model. The combination of this dual‐drug lipoprotein and anti‐PD‐L1 was further shown to increase its antitumor efficiency.^391^

The incorporation of Mn^2+^ or lanthanides and so on into multifunctional lipid‐NPs can significantly promote the activation of STING pathway and anticancer effects.^393^, ^394^, ^395^ For example, Zhu et al.^396^ developed a platform based on a manganese‐containing core with a PEG‐modified PL bilayer shell, where Mn^2+^ has the ability to optimize MRI and activate cGAS–STING pathway. Another interesting article reported that CDN‐Mn^2+^ coordination polymers with a PEGylated lipid layer (DOPC: cholesterol: DSPE‐PEG5000), with an average size of 80–160 nm and a neutral surface charge, retained the good tolerance and safety of liposomes while achieving drug dose savings and minimal side effects by amplifying the activation of the STING pathway.^312^ Compared with soluble CDNs, CDN‐Mn^2+^ NP significantly increased CDN uptake and cytoplasmic internalization by BMDCs, synergistically promoted STING pathway activation, and increased IFN‐β and TNF‐α secretion. Intratumoral or intravenous injection of lipidated CDN‐Mn^2+^ NPs significantly enhanced the tumor accumulation of CDNs and promoted NK‐cell and DC activation in the TME, thereby effectively enhancing the antitumor effects of CDNs in B16F10 and NOOC1 tumor‐bearing mice.^312^ Amorphous porous manganese phosphate NPs‐based PL‐coated hybrid NPs encapsulating with DOX have shown a similar enhancement of cGAS/STING activities, as well as improvement of antitumor immune responses and therapeutic potential.^397^ Furthermore, the Mn^2+^ combined with radiosensitization (several radio‐sensitizers such as hafnium, gold, and lanthanide elements, and those high‐Z metals) is a viable strategy to promote synergistic antitumor activity. Dai and coworkers^398^ reported a novel pH‐responsive lanthanide‐doped radiosensitizer‐based metal‐phenolic network STING agonist named NaGdF4:Nd@NaLuF4@PEG‐polyphenol/Mn (DSPM), which demonstrated a robust anticancer efficiency via an enhanced STING activation in combination with radiotherapy and immunotherapy.

## 6 LIPOSOMALLY SUPPORTED STING AGONISTS IN COMBINATION WITH OTHER THERAPIES

### 6.1 Liposomally supported STING agonists combined with radiotherapy or chemotherapy

Some traditional tumor ablation approaches are highly effective partners for STING agonists. Chemotherapy and radiotherapy have been reported to provide sufficient tumor‐specific neoantigens and TAAs to achieve STING‐agonist‐induced antitumor immunity, which allows the whole tumor to be transformed into a vaccine.^399^ A previous report by Jinjin and colleagues^389^ showed that a 93‐O17S‐F/cGAMP lipidoid promoted antigen presentation of APCs via electrostatic adsorption of DOX‐pretreatment‐induced antigens, thus producing a strong antitumor immune response and increasing survival rates in a B16F10 tumor model, as well as exhibiting excellent immune memory. Notably, DOX and the 93‐O17S‐F/cGAMP lipidoid were administered sequentially in this current system; additional attempts in future studies could be made to explore integrated NP formulations to simplify the handling procedure and improve the therapeutic benefits.^389^

A consensus is that chemotherapy‐ or radiotherapy‐induced cell death can be immunogenic; DAMP or PAMP molecules released from tumor cells after treatments can promote DC activation and maturation.^400^ Moreover, the functional cytosolic–DNA–sensing STING pathway is required for immunogenic radiotherapy‐mediated antitumor immunity, and targeting cGAS‐STING pathway has shown a capacity to improve the therapeutic efficiency of radiotherapy.^401^, ^402^, ^403^ It is tempting to speculate that STING agonists in combination with radiotherapy or chemotherapy may produce a better synergistic antitumor effect, and Gu et al.^404^ indeed showed that the enchantment of antitumor efficiency through liposomal oxaliplatin in combination with localized STING activation. In accordance with these findings, Liu et al.^405^ devised a pH‐sensitive liposomal NP encapsulating cGAMP (liposomal‐NP_cGAMP_), with an average size of around 120 nm and a negative surface charge of about −40 mV. The liposomal NP_cGAMP_ and tumoral APC‐targeted delivery stimulated STING activation, converting the TME from an immunosuppressive state to a proinflammatory state in lung metastases, and improving host sensitivity to radiotherapy. This liposomal NP_cGAMP_, combined with radiotherapy (8 Gy × 3), induced potent anticancer immune responses, effectively delayed the growth of the primary tumors, and improved survival with a reduced number of metastatic lung foci on each lung in B16‐OVA and 4T1 tumor‐bearing mice.^405^ Furthermore, a recent study reported a bio‐liposome composed of intermingled platelet membranes, liposomes with NK‐activatable target antigen (IgG antibodies) and cisplatin pre‐drug. Bioliposome here protected the cisplatin predrug from degradation, enhanced its RES evade and prolonged the half‐life time, as well as improved tumor accumulation of cisplatin predrug. Most interestingly, the liposomal‐activated NK cells can precisely identify and kill the dying tumor cells induced by chemotherapeutic agents, thus leading to an immune‐responsive TME via STING–IFN pathway that further enhanced the immunotherapy effectiveness.^406^

### 6.2 Liposomally supported STING agonists combined with vaccines/immunostimulatory agonists

The liposomally supported neoadjuvant combinatorial strategy is focused on improving and reprogramming APC functions in the TME via the loading of antigens and/or immunostimulatory adjuvants. The latter can activate specific PRRs in the APCs, which play a crucial role in recognizing PAMPs and DAMPs, thereby enhancing the subsequent activation and expansion of naive T cells in the LNs.^407^ A PEGylated liposome consisting of DMPC, DOPC, DOPG, DSPE‐PEG, and cdGMP effectively reduced the systemic (blood) distribution of cdGMP and enhanced its accumulation in LNs (especially in APCs) 15‐fold compared with the levels observed 24 h after cdGMP‐free delivery following subcutaneous injection.^408^ Moreover, in this study, PEGylated liposomes were used as trans‐adjuvants for tumor antigens (OVA) through simultaneous delivery rather than encapsulation in one platform, showing superior synergistic cellular and humoral immune effects to antigen vaccine versus free soluble drugs against cancer.^408^ Atukorale et al.^400^ synthesized a dual immunostimulatory agonist (monophosphoryl‐Lipid A [MPLA] and cdGMP)‐encapsulating neutral LNPs (referred to as immuno‐NPs), with a size of 50 nm, consisting of DOPC, DPPC, 3 mol% mPEG2000‐DSPE, and chloroform. MPLA is a TLR4 agonist that is currently used as a vaccine adjuvant due to its potent modulation of innate immunity.^409^ On the one hand, this systemic liposomal delivery promoted selective and increased uptake of agonists by tumor‐resident APCs in a variety of aggressive mouse tumor models (B16F10, 4T1, and D2.A1), resulting in improved potency and safety profiles of the agents compared with free agonist delivery. On the other hand, however, the dual‐agonist immuno‐NPs increased production of IFN‐β (75‐fold) and TNFα (16‐fold) by APCs compared with single‐agonist NPs as a result of the synergistic effect on the activation of innate immunity through nonoverlapping PRR pathways (the STING pathway and the TLR4 pathway, respectively).^400^ The combination of STING agonists and other immunomodulatory adjuvants through liposomal recipes can induce a more potent innate immune response and re‐educate the immunosuppressive TME.^400^ These findings indicate the effectiveness and safety of liposomal vaccines based on STING agonists, with special potential for the future translation of human cancer vaccines.

### 6.3 Liposomally supported STING agonists combined with mAbs or ICIs

Despite that single STING therapy showed unsatisfactory therapeutic results, combining STING agonists with ICI therapies have shown great potential for inducing synergistic therapeutic responses (Table 3).^410^ The basic concept of STING‐agonist‐based combination strategies is to transform the TME from a noninflammatory, immune‐excluded “cold” state to an inflamed, immunostimulatory “hot” state by enhancing the antitumor responses in the cancer‐immune cycle or reducing the evasive effect towards immune defenses.^411^, ^412^ STING and Type I IFN play a key role in enhancing the cross‐presentation by DCs and subsequential T cell migration and chemotaxis. Importantly, adequate adaptive immune cells, in particular CTLs, are a requisite for effective ICI. In addition, STING pathway‐activation‐mediated IFN secretion can alter tumor immunogenicity by upregulating PD‐L1, IDO, MHC I and calreticulin in tumor cells.^413^, ^414^, ^415^ However, it is imperative to acknowledge that while STING plays a beneficial role in the immune response against cancer, its activation inappropriately may hinder the antitumor immune response.^416^ Upregulated PD‐L1 or IDO expression may lead to immunosuppression and STING treatment failure, especially those with strong tolerogenic responses to DNA and low tumor antigenicity.^417^ Thus, the combination of STING agonists and ICIs is a promising avenue to explore to improve monotherapy in multiple ICI resistance models.^418^, ^419^ For example, combination therapy of STING agonist and anti‐transforming growth factor β/PD‐L1 bispecific antibody YM101 produced robust innate and adaptive antitumor responses in immunotherapy‐resistant cancer models by recruiting immune cells and stimulating their function.^418^

Liposome‐based STING agonists in combination with ICI therapy achieve better anticancer effects by trafficking diverse immune cells into the tumor, reverting the immunosuppressive microenvironment, and overcoming ICI resistance with negligible systemic side effects.^373^, ^377^, ^400^, ^420^, ^421^, ^422^ For instance, the previously described cationic liposome composed by Nakamura et al.^373^ was able to overcome anti‐PD‐1 resistance in mice with metastatic melanoma by increasing the expression of PD‐L1 in cancer cells. This increase in PD‐L1 in cancer cells was due to the production of IFN‐γ by activated systemic NK cells, which were initiated from liver macrophages containing the internalized STING liposomes.^373^ Similarly, a synergistic antitumor effect and improved curative responses were observed in B16F10‐ and BRAF‐mutated murine melanoma models with liposome‐based STING‐PD‐L1 combination therapy, emphasizing the critical role of “exhausted” tumor‐infiltrating CD8^+^ T cells in antitumor action.^421^ Furthermore, a simultaneous combination treatment of anti‐PD‐1 and anti‐CTLA‐4 with STING‐agonist‐containing liposomes effectively reduced the number of pulmonary metastatic nodules in a mouse melanoma lung metastasis model, whereas free cGAMP + anti‐PD‐1 + anti‐CTLA‐4 did not.^420^ Related to this, the delivery protocol needs to be carefully designed, setting parameters of interval time and treatment cycle for STING‐LNP and ICI therapy in order to achieve the best antitumor effects.^423^ Additional studies will be needed to analyze whether the liposomal formulation as a delivery platform for combinational STING therapies can be expanded to other therapeutic approaches that are aimed at amplifying T‐cell function, such as agonistic mAbs against various receptors (e.g., 4‐1BB (CD137), OX40 (CD134), CD28, CD27, etc.), cytokines (IL‐2 and IL‐12), and inhibitors of other immunosuppressive molecules (LAG‐3, T‐cell Ig and mucin‐domain containing‐3, IDO). Taken together, future studies need to explore if liposomal‐delivery‐based combination therapies rather than STING monotherapies can achieve improved therapeutic efficiency.

## 7 CONCLUDING REMARKS AND FUTURE OUTLOOK

In this review, we summarized the latest concepts of various types of lipid‐based nanomaterials for cancer vaccination and TME immunomodulation. Ideal lipid‐NP formulations for immunopharmaceuticals must possess characteristics such as biocompatibility, biodegradability, nonimmunogenicity, and targeted delivery to the TME. Lipid‐NPs‐supported cancer vaccines are more effective than “free” drugs to produce a longer‐lasting and broader immune response, inhibit tumor growth and prevent tumor metastasis or recurrence. In addition, TME modulation supported by lipid‐NPs offers more possibilities to remodel the immune profile of TME and assist tumor‐infiltrating immune cells to be effective within the tumor. Specifically, lipid‐NPs act as material carriers effectively protects immunotherapeutic proteins and nucleic acids from degradation, increase the targeting and bioavailability of encapsulated cytokines and genes, achieve spatiotemporally controlled release of immunotherapeutic agents, and effectively reduce systemic side effects of mAbs. In addition, lipid‐NPs are generally considered to be more safe and less immunogenic compared with other delivery vehicles such as polymeric NPs and inorganic NPs. This can be explained by their similarity in composition to cell membranes, which makes them more “natural” to the immune system and less likely to elicit significant immune responses. Therefore, the introduction of lipid‐NPs can amplify immunotherapeutic efficacy while overcoming the current drawbacks of immunotherapy (e.g., poor delivery efficiency, lack of tumor targeting, and severe side effects) to a certain extent. The above‐mentioned advantages have made lipid‐NPs as drug carriers more attractive for cancer immunotherapy.

Furthermore, the STING pathway, which serves as a sensor for initiating the innate immune response, has been identified as a promising target in the treatment of cancer. More basic research with the aim of more deeply understanding the mechanisms of STING signaling and its interaction with TME‐associated factors is necessary to amplify the anticancer potential of STING pathway targeting. It is of note that STING pathway‐activation‐induced local inflammation and antitumor immunity contribute to tumor control in most cases. Still, it is unclear whether chronic inflammation due to long‐term STING agonist exposure promotes tumor growth and metastasis, indicating the importance of balance between STING‐agonist‐induced immune response and inflammation. Therefore, a comprehensive understanding of the biphasic role of STING pathway activation in curbing cancer progression is necessary, and clearer treatment protocols and availability of patient‐selective biomarkers for individual tumor types able to indicate which those patients for whom STING activation will be therapeutically beneficial are still needed.

By discussing the application and potential of lipid‐NPs for STING agonists, we summarized the future perspectives focused on the following points to further promote the application of lipid‐NPs in cancer immunotherapy: (1) in certain instances, lipid‐NPs may trigger an inflammatory response, particularly when CLDs interact with serum proteins and the degradation of biomaterials like LPHNPs. Thus, it is crucial to comprehend the regulation of toxicity and inflammatory responses of lipid‐NPs on immune cells and to enhance immunotherapeutic efficacy and prevent immunotoxicity. (2) The highly heterogeneous and changeable TME is another major challenge that needs to be overcome. Thus, depending on the patient's immune status and tumor characteristics, it would be informative to rationally design lipid‐NPs to carry patient‐specific antigens, appropriate immunotherapeutic agents or combinations thereof; as well as optimize the lipid‐NP parameters (e.g., material composition, geometry, surface chemistry, charge, and mechanical properties) to achieve higher encapsulation rates and controlled release abilities and prompt the lipid‐NPs to cross biological obstacles. (3) Nanopharmaceutical formulations depending on EPR effects remain insignificant effectiveness and exists a potential risk of off‐target effects. Therefore, there should be continued exploration of specific cell–drug interactions in TME to improve the target effects of lipid‐NPs. The combination of immune lipid‐NPs with ablative therapies (chemotherapy, PDT, and radiation therapy) that transiently enhance the permeability of tumor tissue should be strengthened for the enhancement of the intratumor accumulation of NPs to synergistically augment anticancer efficacy. (4) There exists an urgent requirement to continuously identify tumor‐specific and immunogenic neoantigens, innovate and improve existing immunotherapeutic modalities within the domain of fundamental medicine and biology. (5) Further research domain of immune lipid‐NPs can be all‐in‐one platform for diagnosis, treatment and real‐time monitoring through the integration of appropriate monitoring biosensors, enabling the adjustment of drug dosage as necessary. (6) Accurate drug release control and multifunction of complex NPs structures are required, leading to expensive assembly costs for scale‐up production and slow approval from US FDA. Thus, the ratio between efficacy gains and overall cost‐effectiveness including preparation and storage maintenance of the lipid‐NPs must be evaluated. Additionally, programmed preparation processes remain a crucial aspect to be addressed remains a crucial aspect to be addressed to ensure sufficient quantities for clinical applications and consistency between production batches and the safety and quality control of LNP manufacturing.

In conclusion, research on lipid‐NPs‐based cancer immunotherapy is flourishing but there are still some challenges remaining for transition of lipid‐NPs from experimental studies to clinical applications. This review discusses the characteristics of lipid‐NPs subtypes, their applications, as well as their specific strengths and weaknesses. It also takes STING agonists as an example to illustrate the utilization and obstacles of lipid‐NPs in the field, underscoring the imperative for additional investigation to effectively regulate and refine the manipulation of these NPs for improved cancer immunotherapy.

## AUTHOR CONTRIBUTION

*Conceptualization*: H. Y.; *writing—original draft preparation*: H. Y.; *writing—review and editing*: H. Y., Z. G., Z. H. Z., and W. D. R.; *figure—original draft preparation*: H. Y. and J. Z. H.; *figure—visualization*: H. Y.; *supervision*: W. D. X. and P. t. D.; *final editing*: P. t. D. All authors have read and approved the final manuscript.

## CONFLICT OF INTEREST STATEMENT

The authors declare no conflict of interest.

## ETHICS STATEMENT

Not applicable.

## Data Availability

Not applicable.
